# Oral administration of TiO_2_ nanoparticles during early life impacts cardiac and neurobehavioral performance and metabolite profile in an age- and sex-related manner

**DOI:** 10.1186/s12989-021-00444-9

**Published:** 2022-01-05

**Authors:** Ninell P. Mortensen, Wimal Pathmasiri, Rodney W. Snyder, Maria Moreno Caffaro, Scott L. Watson, Purvi R. Patel, Lakshmi Beeravalli, Sharmista Prattipati, Shyam Aravamudhan, Susan J. Sumner, Timothy R. Fennell

**Affiliations:** 1 Discovery Sciences, RTI International, 3040 E Cornwallis Road, Research Triangle Park, NC 27709 USA; 2 UNC Nutrition Research Institute, The University of North Carolina at Chapel Hill, 500 Laureate Way, Kannapolis, NC 28081 USA; 3 Joint School of Nanoscience and Nanoengineering, 2907 East Gate City Blvd., Greensboro, NC 27401 USA

**Keywords:** TiO_2_ NP, Early life exposure, Pre-weaning rats, Cardiac performance, Neurobehavioral assessment, Neurotransmitters, Metabolomic analysis

## Abstract

**Background:**

Nanoparticles (NPs) are increasingly incorporated in everyday products. To investigate the effects of early life exposure to orally ingested TiO_2_ NP, male and female Sprague–Dawley rat pups received four consecutive daily doses of 10 mg/kg body weight TiO_2_ NP (diameter: 21 ± 5 nm) or vehicle control (water) by gavage at three different pre-weaning ages: postnatal day (PND) 2–5, PND 7–10, or PND 17–20. Cardiac assessment and basic neurobehavioral tests (locomotor activity, rotarod, and acoustic startle) were conducted on PND 20. Pups were sacrificed at PND 21. Select tissues were collected, weighed, processed for neurotransmitter and metabolomics analyses.

**Results:**

Heart rate was found to be significantly decreased in female pups when dosed between PND 7–10 and PND 17–20. Females dosed between PND 2–5 showed decrease acoustic startle response and when dosed between PND 7–10 showed decreased performance in the rotarod test and increased locomotor activity. Male pups dosed between PND 17–20 showed decreased locomotor activity. The concentrations of neurotransmitters and related metabolites in brain tissue and the metabolomic profile of plasma were impacted by TiO_2_ NP administration for all dose groups. Metabolomic pathways perturbed by TiO_2_ NP administration included pathways involved in amino acid and lipid metabolism.

**Conclusion:**

Oral administration of TiO_2_ NP to rat pups impacted basic cardiac and neurobehavioral performance, neurotransmitters and related metabolites concentrations in brain tissue, and the biochemical profiles of plasma. The findings suggested that female pups were more likely to experience adverse outcome following early life exposure to oral TiO_2_ NP than male pups. Collectively the data from this exploratory study suggest oral administration of TiO_2_ NP cause adverse biological effects in an age- and sex-related manner, emphasizing the need to understand the short- and long-term effects of early life exposure to TiO_2_ NP.

**Supplementary Information:**

The online version contains supplementary material available at 10.1186/s12989-021-00444-9.

## Background

There is a significant knowledge gap in understanding the biological impact of early life exposure to nanoparticles (NPs). Early life represents a vulnerable window because perturbations in temporally sequenced developmental processes may have long-term health impacts. Oral exposure to NPs through diet and numerous other sources [1–7] are unavoidable, which reinforces the necessity to understand the short- and long-term risk for health outcome. Food grade particulate TiO_2_ is used as a white pigment and brightening agent and has been reported in food at a range of concentrations [8] with between 17–74% on nanoscale (< 100 nm) [1, 9–12]. Furthermore, TiO_2_ particles have been detected in 7 out of 15 adult (ages 56–104) liver samples (collected postmortem) [13], suggesting oral uptake of TiO_2_ particles. Regarding exposure in children, the estimated daily consumption of TiO_2_ is between 0.2–1.9 mg/kg body weight (bw) per day for infants, 0.6–9.2 mg/kg bw/day for toddlers, and 0.9–10.4 mg/kg bw/day for children [14]. While TiO_2_ is classified as a Generally Regarded As Safe (GRAS) material in both the USA and EU, concerns regarding TiO_2_ NP are increasing, to the point that France banned TiO_2_ in food products starting January 2020 [15, 16], and the European Food Safety Authority in May 2021 announced that TiO_2_ E171 is no longer considered a safe food additive [17]. While orally administered TiO_2_ NP has been studied in adult animal models [18–22], little is known about the biological impact of TiO_2_ NP and potential toxicity in developing animals at different pre-weaning ages and developmental stages.

Studies of early life exposure to NPs in pre-weaning rats are few [23–27]. Aluminum oxide (Al_2_O_3_) NP orally administered to male and female rat pups between PND 17–20 at a concentration of 10 mg/kg bw resulted in increased liver-to-body weight (bw) ratio in male pups and changes in concentration of neurotransmitters and related metabolites in the brain for both male and female pups [26]. Neurotoxic effects, including cerebellar ataxia-like symptoms, dysfunction of motor coordination, and impairment of locomotor activity were reported in rats dosed with silver nanoparticles (Ag NPs) by intranasal instillation for 14 consecutive weeks, starting in neonatal rats [25]. Food grade TiO_2_ E171 and copper oxide (CuO) NPs orally administered to male and female pups between PND 7–10 caused an increase in immune cells in both the small and large intestinal tract [28]. To the authors’ knowledge, no study has investigated the biological responses to TiO_2_ NPs following oral administration in male and female rat pups at different pre-weaning ages.

Organs are formed during the fetal stage, continue to develop anatomically and physiologically during early life, and mature during adolescence. These organs include the gastrointestinal tract [29–33], the brain and the central nervous system [34–36], and the heart [37, 38]. The gastrointestinal tract undergoes significant development, and the stomach of neonates and juveniles has considerable anatomical and physiological differences compared to that of adult rats [39]. The histology of rat stomachs at PND 4 shows no secretory cells and has a neutral pH [40]. At PND 7, very little development has occurred. At PND 14, development has led to cellular differentiation in the gastric glans, Parietal cells, and deeper in the mucosa the basophilic chief cells are apparent [40]. Functionally the stomach is still largely immature at PND 14, with minimal pepsinogen and acid secretion, and pH starts to drop. Antral gastrin secretion increases dramatically from PND 18–20, and pH drops further. At PND 21, the mucosal cell population is comparable to that of the adult stomach. Significant acid secretion and pepsinogen activation happens around weaning, and at PDN 28 the adult anatomy of the stomach is in place, while final maturation takes place around 6 weeks after birth [41]. The anatomical development of the stomach and gastrointestinal tract may impact NP transformation and uptake differently than in adult animals.

This study was conducted as part of National Institute of Environmental Health Sciences (NIEHS) Nanomaterials Health Implications Research (NHIR) Consortium, and the investigated TiO_2_ NP was provided by the NIEHS-NHIR Consortium. In this study, male and female rat pups were orally administered TiO_2_ NP at three different ages prior to weaning. The purpose of this study was to investigate the biological effects of TiO_2_ NPs, and therefore animals were dosed with TiO_2_ P25, rather than the food grade TiO_2_ E171 of which between 17–74% is estimated to be on the nanoscale (< 100 nm) [1, 10–12]. However, both TiO_2_ P25 and TiO_2_ E171 are spherical particles with similar crystallinity. The oral route was chosen to investigate ingestion of TiO_2_ NPs. At the two earliest dosing ages (PND 2–5 and PND 7–10) the pups are solely ingesting the dam’s milk, while at the later dosing age (PND 17–20) the pups, while still relying on the dam’s milk, have started to eat solid feed. The gastrointestinal tract has not yet reached the adult anatomical and physiological stages at the three pre-weaning dosing ages [42]. In this study four consecutive daily doses of 10 mg/kg bw were administered. The dose selection was guided by the literature of TiO_2_ concentrations reported in food [8, 14]. TiO_2_ E171 has been found in food at a concentration between 0.02–9.0 mg TiO_2_/g product [43] with an estimated daily consumption of TiO_2_ as high as 10.4 mg/kg bw for children [14]; for this reason we chose to administer 10 mg/kg bw/day of TiO_2_ NP for four consecutive days. The body weight of the pups was recorded from the start of dosing until the termination of the study. Utilizing non-invasive methods, basic cardiac performance and neurobehavioral assessments were performed. Following termination at PND 21, organ-to-bw ratio was calculated for liver and brain, the concentration of monoamine neurotransmitters and related metabolites was determined in brain tissue, and metabolomic analysis was performed on plasma. The motivation behind evaluating cardiac performance arose from findings that the cardiovascular system was particularly sensitive to the effects of nanomaterials in adult animals [44–47], and that early life exposure to particulate matter in polluted air increases adult susceptibility to cardiac dysfunction [48, 49]. Liver-to-bw ratio is a predictive endpoint for accurately detecting organ toxicity [50], and since the liver is the first organ receiving blood coming from the intestinal tract, filtering, and detoxifying xenobiotics, oral administered NPs may therefore impact hepatic health and functions. Here neurobehavioral assessment, and quantification of neurotransmitters and related metabolites in brain tissue were conducted to explore possible interference of the bidirectional communication between the gut and the brain (gut-brain axis) following oral TiO_2_ NP exposure. The bidirectional communication between the gut and the brain is receiving increasing interest, as are the consequences of interference with this axis, which has been implicated in a wide range of functional and inflammatory disorders, obesity, and eating disorders [51, 52].

This exploratory study is the first report of TiO_2_ NP administered orally to male and female rat pups at three different pre-weaning ages and investigates the biological effects of TiO_2_ NPs exposure during early life as a single dose study. The presented study is not intended for dose response assessment, risk assessment, or no observed adverse effect level determination. The results presented here are for basic understanding of the biological effects of orally administered TiO_2_ NP in rat pups at three pre-weaning age ranges, and the roles of age and sex in early life exposure at different developmental stages.

## Results

### In vitro simulated gastric digestion of TiO_2_ NPs

The stability and hydrodynamic diameter of TiO_2_ NPs were evaluated following simulated gastric digestion for three different pre-weaning ages: PND 7, PND 14, and PND 21 at three different time points (1, 2, and 4 h). Inductively coupled plasma - optical emission spectrometry (ICP-OES) analysis of digested TiO_2_ NPs did not show any dissolution, while the hydrodynamic diameter and polydispersity index (PdI) increased showing NP aggregation (Additional file 3: Table S1). Scanning electron microscope (SEM) images of digested TiO_2_ NPs confirmed the findings of aggregation and no dissolution (Additional file 1: Figure S1). The simulated digestion data suggests that the gastrointestinal environment in developing rats is likely to cause NP aggregation but not dissolution of TiO_2_ NP.

### Dosing solution characterization and stability

**Fig. 1:**
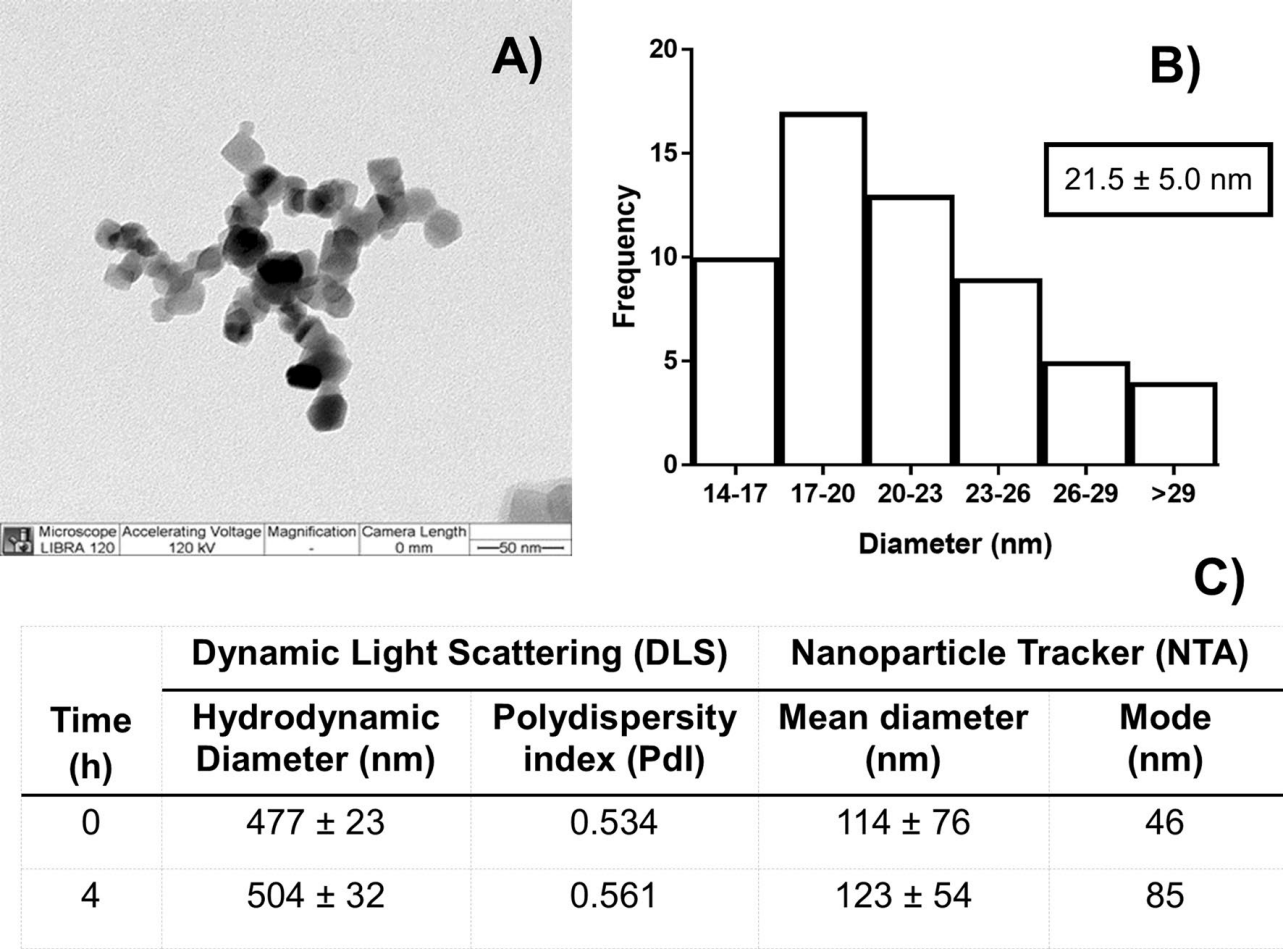
**A** TEM of TiO_2_ NP and **B** histogram of size distribution. **C** Characterization of the 2 mg/mL dosing solutions was conducted by DLS and NTA at 0 and 4 h after preparation

The TiO_2_ NP provided by the NIEHS-NHIR Consortium was characterized by transmission electron microscopy (TEM), which showed a diameter of 21.5 ± 5.0 nm of the pristine NPs (Fig. 1A, B). The TiO_2_ NP dosing solution was formulated at 2 mg/mL in deionized water and characterized by dynamic light scattering (DLS) and nanoparticle tracking analysis (NTA) showing a diameter of 477 ± 23 nm and 114 ± 76 nm, respectively (Fig. 1C). The PdI for TiO_2_ NP indicated some aggregation but increasing sonication time did not lower the diameter or the PdI. The dosing solution was stable for 4 h.

### Body weight (bw) and organ-to-bw ratio

**Fig. 2:**
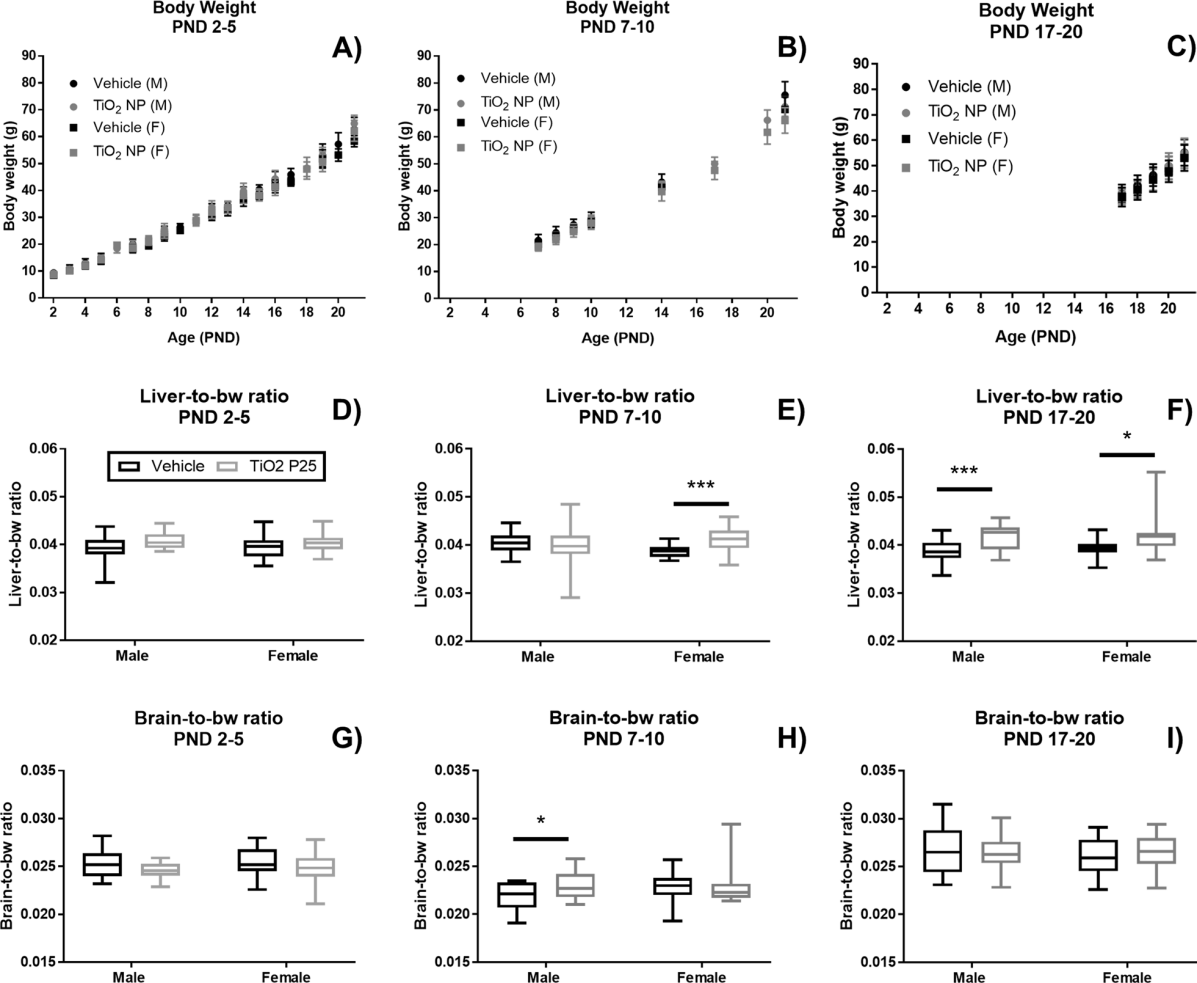
Body weight (bw) (**A**–**C**), liver-to-bw ratio (**D**–**F**), and brain-to-bw ratio (**G**–**I**) were measured for male (n = 15) and female (n = 15) rat pups orally dosed with TiO_2_ NP and vehicle control between PND 2–5 (**A**, **D**, **G**), PND 7–10 (**B**, **E**, **H**), and PND 17–20 (**C**, **F**, **I**). No changes in bw were observed as a result of TiO_2_ NP administration. TiO_2_ NP led to increased liver-to-bw ratio at PND 21 for female pups dosed between PND 7–10 and PND 17–20, while liver-to-bw ratio increased for male pups dosed between PND 17–20. Male pups dosed between PND 7–10 had significantly increased brain-to-bw ratio. Body weight is presented as mean ± standard deviation. Box and whisker plots of organ-to-bw ratio data where the box extends from the 25th to 75th percentile and shows the median value, while the whiskers show the minimum and maximum value. TiO_2_ NP dose groups are shown in black and vehicle control in gray. Statistical analyses were conducted using Mann–Whitney U test: *P-value < 0.05, **P-value < 0.01, ***P-value < 0.005

The bw for male and female pups was recorded for the three TiO_2_ NP dosing groups, from the day the first dose was administered and until the pups were sacrificed at PND 21. No changes in bw were observed as a result of TiO_2_ NP administration (Fig. 2A–C). However, TiO_2_ NP administered between PND 7–10 led to significantly increased liver-to-bw ratio in female pups and when administered between PND 17–20 the increase was statistically significant for both male and female pups (Fig. 2D–F). The brain-to-bw ratio was significantly increased for male pups dosed between PND 7–10 withTiO_2_ NP (Fig. 2G–I).

### Cardiac assessment

**Fig. 3:**
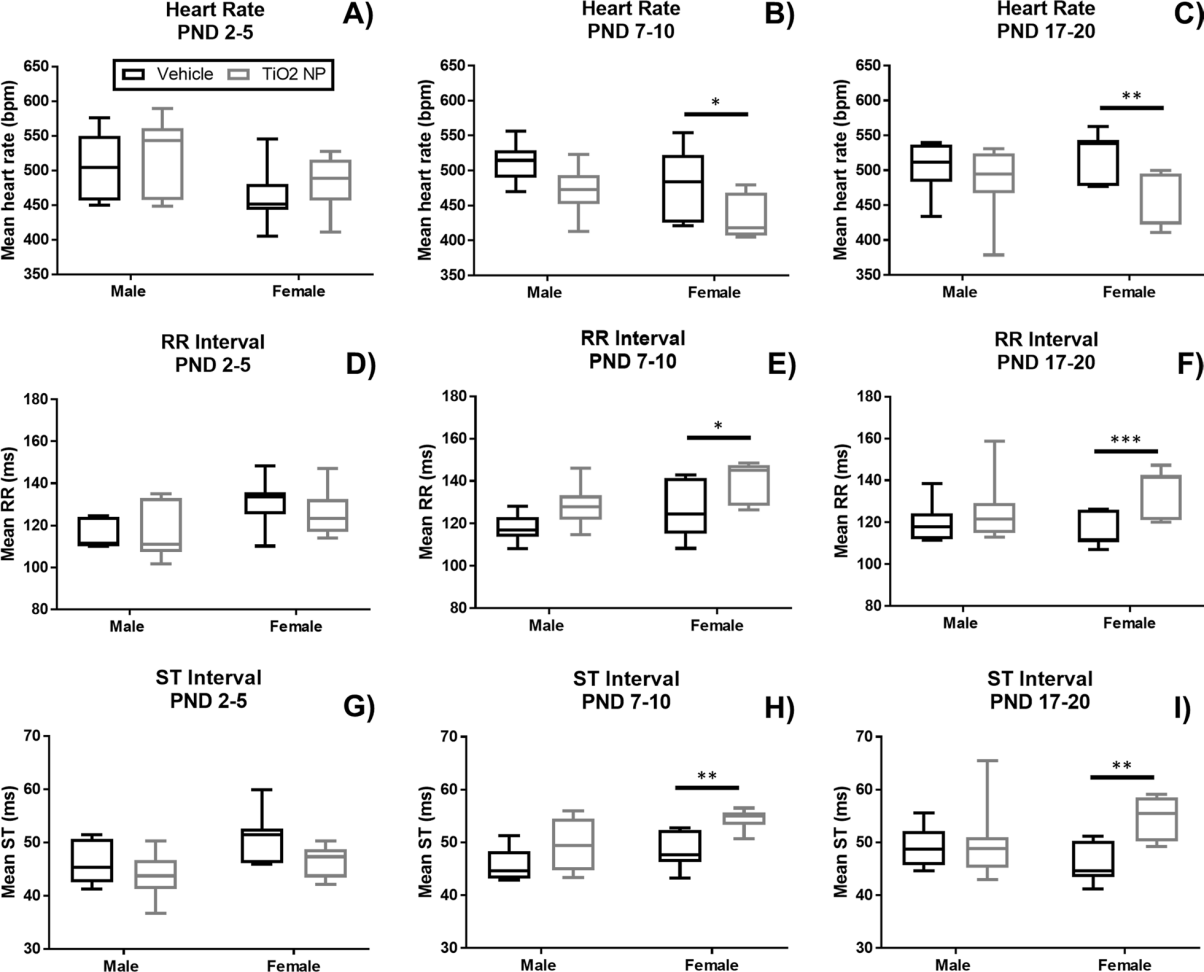
Cardiac assessment at PND 20 for male (n = 6–7) and female (n = 6–7) pups dosed between PND 2–5 (**A**–**C**), PND 7–10 (**D**–**F**), and PND 17–20 (**G**–**I**). Oral administration of TiO_2_ NP between PND 7–10 and PND 17–20 resulted in significantly decreased heart rate for female pups, and significantly increased ST and RR intervals. No significant changes in cardiac performance were found for male pups dosed with TiO_2_ NP. Box and whisker plots, where the box extends from the 25th to 75th percentile and shows the median value, while the whiskers show the minimum and maximum value. TiO_2_ NP dose groups are shown in black and vehicle control in gray. Statistical analyses were conducted using Mann–Whitney U test: *P-value < 0.05, **P-value < 0.01, ***P-value < 0.005

The electrocardiogram (ECGs) of unrestrained and awake pups were measured non-invasively at PND 20. TiO_2_ NP administered between PND 7–10 and PND 17–20 changed the heartbeat in female pups, resulting in significantly decreased heart rate (Fig. 3A–C) and significantly increased RR interval (Fig. 3D–F) at PND 20. The ST segment, which is the interval between ventricular depolarization and ventricular repolarization, was significantly increased (Fig. 3G–I). TiO_2_ NP had no significant impact on the ECGs in male pups, although a decrease in heart rate was observed for male pups dosed between PND 7–10 (Fig. 3B).

### Basic neurobehavioral assessment

**Fig. 4:**
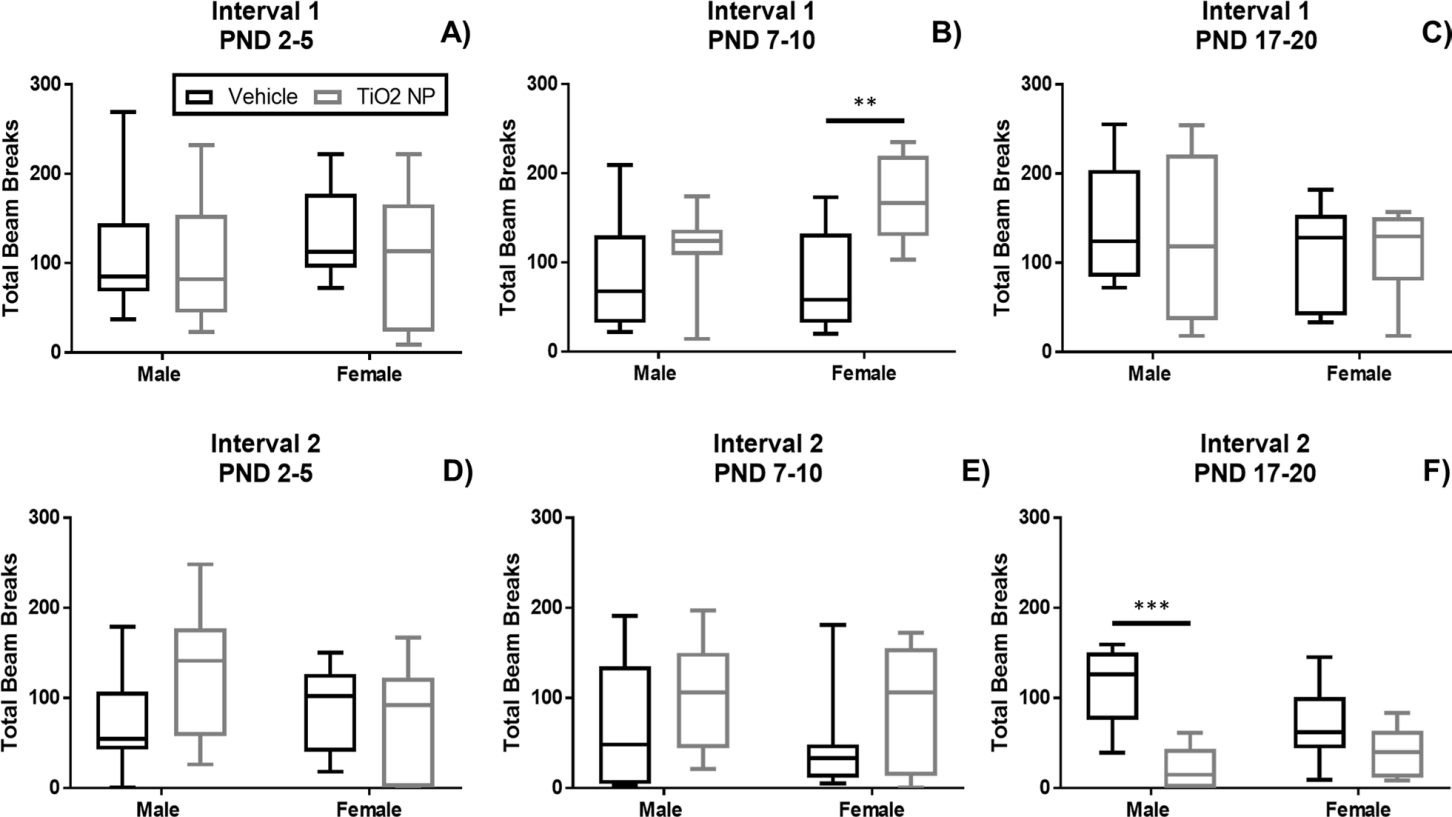
Locomotor activity was measured at PND 20 at two intervals: Interval 1 (0–5 min) and Interval 2 (> 5–10 min) for male (n = 8) and female (n = 8) pups dosed between PND 2–5 (**A**, **D**), PND 7–10 (**B**, **E**), and PND 17–20 (**C**, **F**). The locomotor activity was significantly increased for Interval 1 for female pups dosed between PND 7–10, while the locomotor activity was significantly decreased for male pups in Interval 2 dosed between PND 17–20. Box and whisker plots, where the box extends from the 25th to 75th percentile and shows the median value, while the whiskers show the minimum and maximum value. TiO_2_ NP dose groups are shown in black and vehicle control in gray. Statistical analyses were conducted using Mann–Whitney U test: *P-value < 0.05, **P-value < 0.01, ***P-value < 0.005

**Fig. 5:**
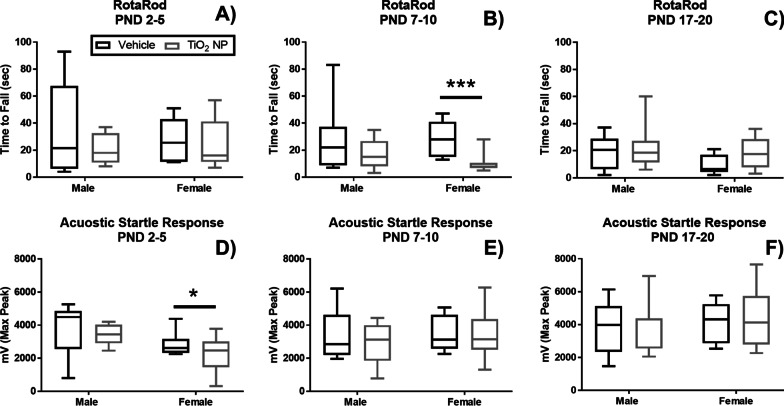
RotaRod performance (**A**–**C**) and acoustic startle response (**D**–**F**) where recorded on PND 20 for male (n = 8) and female (n = 8) pups dosed between PND 2–5 (**A**, **D**), PND 7–10 (**B**, **E**), and PND 17–20 (**C**, **F**). Female pups dosed with TiO_2_ NP between PND 7–10 showed significantly decreased performance in the RotaRod test, while female pups dosed between PND 2–5 showed decreased response in the acoustic startle response test. Box and whisker plots where, the box extends from the 25th to 75th percentile and shows the median value, while the whiskers show the minimum and maximum value. TiO_2_ NP dose groups are shown in black and vehicle control in gray. Statistical analyses were done using Mann–Whitney U test: *P-value < 0.05, **P-value < 0.01, ***P-value < 0.005

Locomotor activity (Fig. 4), rotarod (Fig. 5A–C), and acoustic startle response (Fig. 5D–F) were assessed at PND 20 for all three dosing groups. Locomotor activity was measured in two intervals, Interval 1 (0–5 min) and Interval 2 (> 5–10 min). Locomotor activity significantly increased in Interval 1 in female pups administered TiO_2_ NP between PND 7–10 (Fig. 4B)**;** however, male pups dosed with TiO_2_ NP between PND 17–20 led to a significant decrease of locomotor activity in Interval 2 (Fig. 4F). TiO_2_ NP administration led to a significant decrease in rotarod performance for female pups dosed between PND 7–10 (Fig. 5B). Female pups dosed with TiO_2_ NP between PND 2–5 had a significantly decreased acoustic startle response at PND 20 (Fig. 5D).

### Concentrations of neurotransmitters and related metabolites in brain

The concentrations of six monoamine neurotransmitters and related metabolites connected to memory, emotion, depression, anxiety, and neuroendocrine function were quantified in the brain of male and female pups. The neurotransmitter dopamine (DA) and its metabolites dihydroxyphenylacetic acid (DOPAC), homovanillic acid (HVA), and norepinephrine (NE), as well as the neurotransmitter serotonin (5-HT) and its primary metabolite 5-hydroxyindole-3-acetic acid (5-HIAA) were determined in the right brain half for male and female pups for the three dosing groups (Table 1). The concentration of DA was significantly increased in the brains of both male and female pups dosed between PND 2–5. In both male and female pups dosed with TiO_2_ NP between PND 7–10 the concentration of DOPAC was significantly increased and HVA was significantly decreased, while DA concentration was significantly increased only in females. In both male and female pups dosed between PND 17–20 HVA was significantly decreased, while NE was only significantly decreased in female pups. The concentrations of 5-HT and 5-HIAA were not altered by TiO_2_ NP exposure.

**Table 1:** Concentration (ng/g brain tissue) of neurotransmitters and related metabolites in brain tissue (n = 7–8)

Age at administration	PND 2–5		PND 7–10		PND 17–20	
	Vehicle	TiO_2_ NP	Vehicle	TiO_2_ NP	Vehicle	TiO_2_ NP
*Male*						
DA^2^	483 ± 29.7^1^	529 ± 34.4*^3^	507 ± 57.6	530 ± 44.0	493 ± 43.6	423 ± 57.1
DOPAC	129 ± 11.3	134 ± 11.2	111 ± 12.0	149 ± 12.8***	122 ± 14.3	111 ± 17.9
HVA	147 ± 15.3	147 ± 23.5	205 ± 48.1	154 ± 13.6*	288 ± 100	159 ± 47.9*
NE	247 ± 18.6	251 ± 38.4	242 ± 29.8	257 ± 34.9	235 ± 34.1	198 ± 56.6
5-HT	238 ± 57.0	239 ± 60.2	239 ± 47.3	283 ± 28.8	274 ± 52.6	276 ± 36.1
5-HIAA	463 ± 155	489 ± 71.4	471 ± 124	458 ± 19.4	420 ± 50.7	487 ± 78.4
*Female*						
DA	485 ± 28.4	544 ± 41.1***	495 ± 30.2	549 ± 39.1***	508 ± 37.8	466 ± 78.3
DOPAC	127 ± 9.71	135 ± 13.5	116 ± 8.71	149 ± 24.5*	123 ± 7.04	127 ± 26.2
HVA	143 ± 12.0	147 ± 23.5	207 ± 16.2	154 ± 13.4***	266 ± 94.4	172 ± 37.8**
NE	264 ± 40.2	255 ± 37.1	239 ± 33.8	264 ± 29.7	305 ± 26.0	186 ± 44.5***
5-HT	242 ± 46.0	310 ± 68.6	249 ± 48.7	284 ± 27.2	317 ± 41.2	261 ± 46.5
5-HIAA	498 ± 58.4	516 ± 74.9	530 ± 79.9	442 ± 61.7	443 ± 42.9	477 ± 47.1

^1^Data is presented as median ± standard deviation

^2^Dopamine (DA), dihydroxyphenylacetic acid (DOPAC), homovanillic acid (HVA), norepinephrine (NE) (also called noradrenaline (NA) or noradrenalin), serotonin (5-HT), and 5-hydroxyindole-3-acetic acid (5-HIAA)

^3^Significant differences in concentrations between TiO_2_ NP and the corresponding vehicle control is indicated: *P < 0.05, **P < 0.01, and ***P < 0.005

### Metabolomic analysis of plasma

**Fig. 6:**
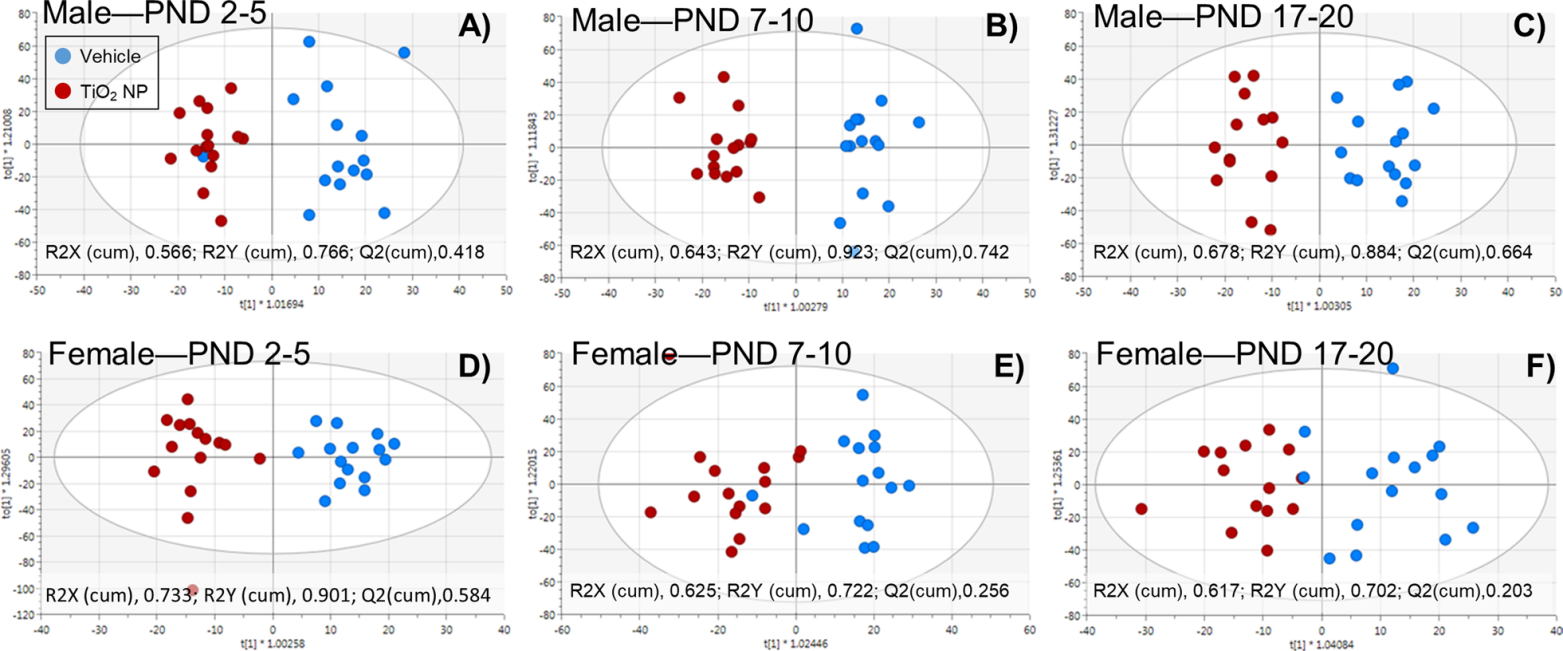
OPLS-DA score plots using (R2X, R2Y, and Q2) plasma metabolite differentiating male (**A**, **C**, **E**) and female (**B**, **D**, **F**) pups orally administered TiO_2_ NP from vehicle control. Rat pups were dosed between PND 2–5 (**A**, **D**), PND 7–10 (**B**, **E**), or PND 17–20 (**C**, **F**) and sacrificed on PND 21. OPLS-DA plots show a clear grouping of metabolites in male pups (**A**–**C**). However, for female pups OPLS-DA plots show a clear grouping of metabolites in female pups for PND 2–5 (**D**), but not for PND 7–10 (**E**) and PND 17–20 (**F**)

A total of 186 metabolites belonging to different metabolite classes: 40 acylcarnitines, 21 amino acids, 1 monosaccharide, 90 glycerophospholipids, 15 sphingolipids, and 21 biogenic amines, were quantified in plasma collected at PND 21. In addition, 44 metabolite sums and ratios (metabolite indicators) were calculated. A supervised orthogonal partial least squares discriminate analysis (OPLS-DA) showed good differentiation between TiO_2_ NP and vehicle exposed male pups for all three dose ages (Fig. 6A–C), while the OPLS-DA showed good differentiation for female pups dosed between PND 2–5 (Fig. 6D), there was slight overlap for female pups dosed between PND 7–10 (Fig. 6E) and PND 17–20 (Fig. 6F). For all six dosing groups amino acids were the largest class of metabolites with VIP ≥ 1 in the OPLS-DA, while glycerophospholipids was the largest class of significantly changed metabolites between dosed groups and control with a P-value ≤ 0.05 (Additional file 2: Figure S2). Metabolites with P-value ≤   0.05 and/or VIP ≥ 1 in the OPLS-DA for male and female pups are listed in Additional files 4 and 5: Tables S2 and S3, respectively.

The metabolite with the highest fold change (FC) between TiO_2_ NP and vehicle group was acetylornithine (male, PND 7–10: 1.82; male, PND 17–20: 1.66; female, PND 7–10: 1.86; female, PND 17–20: 1.99). Another metabolite with high FC was sulfoxidized methionine (Met-SO) (male, PND 7–10: 1.49; male, PND 17–20: 1.62; female, PND 7–10: 1.65; female, PND 17–20: 1.42). The ratio of Met-SO to the unmodified methionine (Met) pool indicates systemic oxidative stress [53] and was observed for pups dosed between PND 7–10 (male: 1.08; female: 1.25) and PND 17–20 (male: 1.27; female: 1.28). In addition, there were some metabolites that showed higher statistical significance and/or higher VIP values (e.g., alanine, arginine, citrulline, glutamine, glycine, lysine, proline, acyl carnitines, glycerophospholipids, and sphingolipids (see Additional files 4 and 5: Tables S2 and S3 for details)).

Pathway analysis in MetaboAnalyst software (using both small molecule metabolites and lipid metabolites separately) was conducted to evaluate perturbations of metabolic pathways upon TiO_2_ NP exposure based on the biochemical profile of plasma (Tables 2 and 3, respectively). The metabolomics pathway analysis showed a high level of overlap in the significantly perturbed pathways between dose groups, being amino acid metabolism and biosynthesis as well as aminoacyl-tRNA biosynthesis for all three dosing ages and both sexes. In addition, pathway perturbations in lipid subclasses impacted by TiO_2_ NP administration were also observed in the enrichment analysis (Table 3).

**Table 2:** Pathway analysis results (FDR correct P-values) using MetaboAnalyst showing the enriched metabolic pathways perturbed upon TiO_2_ NP exposure

Metabolic pathway	PND 2–5		PND 7–10		PND 17–20	
	Male	Female	Male	Female	Male	Female
Alanine, aspartate, and glutamate metabolism	–^1^	–	8.06 × 10^−3^	–	–	–
Aminoacyl-tRNA biosynthesis	1.95 × 10^−8^	1.90 × 10^−7^	1.53 × 10^−8^	3.02 × 10^−9^	8.04 × 10^−6^	0.00184
Arginine and proline metabolism	0.00222	0.0342	5.33 × 10^−3^	–	0.0165	6.90 × 10^−4^
Arginine biosynthesis	0.0293	0.00599	4.19 × 10^−4^	–	1.14 × 10^−4^	1.00 × 10^−4^
beta-Alanine metabolism	–	–	0.0206	–	–	–
d-Arginine and d-ornithine metabolism	0.0293	–	–	–	–	–
d-Glutamine and d-glutamate metabolism	0.00344	–	–	–	0.0439	–
Glutathione metabolism	0.0293	–	0.0446	–	–	–
Phenylalanine, tyrosine, and tryptophan biosynthesis	–	–	0.0446	–	–	–

^1^FDR corrected P-value > 0.05 was marked by “–”,

**Table 3:** Results (FDR correct P-values) of Metabolite Set Enrichment Analysis using MetaboAnalyst showing consistent changes among lipids

Lipid subclass	PND 2–5		PND 7–10		PND 17–20	
	Male	Female	Male	Female	Male	Female
1-Alkyl,2-acylglycerophosphocholines	0.00119	2.02 × 10^−37^	1.82 × 10^−15^	6.29 × 10^−9^	0.0014	4.21 × 10^−4^
Acylcarnitines	–^1^	–	0.00779	8.72 × 10^−4^	–	–
Ceramide phosphocholines	–	–	7.41 × 10^−4^	–	8.90 × 10^−4^	–
Diacylglycerophosphocholines	6.88 × 10^−60^	8.84 × 10^−142^	6.13 × 10^−72^	2.74 × 10^−5^	6.38 × 10^−71^	4.81 × 10^−34^
Fatty acid esters	–	–	–	0.0306	–	–
Fatty acylcarnitines	0.0445	0.00391	1.07 × 10^−9^	2.52 × 10^−14^	–	2.77 × 10^−4^
Glycerophosphocholines	–	1.59 × 10^−4^	0.00779	–	0.0495	–
Glycerophospholipids	–	3.92 × 10^−5^	0.00779	0.0460	–	–
Lysophosphatidylcholines	0.0420	5.75 × 10^−5^	–	0.0126	0.0373	0.0171
Monoacylglycerophosphocholines	–	0.0439	0.0162	–	–	–
Phosphosphingolipids	1.13 × 10^−5^	7.19 × 10^−5^	1.30 × 10^−10^	–	0.00354	0.00155
Short-chain acylcarnitines	–	–	6.11 × 10^−8^	1.30 × 10^−9^	–	2.87 × 10^−9^

^1^FDR corrected P-value > 0.05 was marked by “–”

## Discussion

The purpose of this exploratory study was to investigate the biological effects of TiO_2_ NPs following oral exposure during early life. Pre-weaning male and female rat pups were orally dosed with TiO_2_ NPs at three different ages to investigate the biological responses to TiO_2_ NP exposure. Investigations of TiO_2_ NP exposure in postnatal animals are fundamental for understanding the potential health risk of ingestion during early life.

During postnatal development the gastric pH drops and enzyme secretion increases [40]. Simulated gastrointestinal digestion of NP in vitro suggests that digestion may dramatically change the physiochemical properties of NPs [54–57]. Here, in vitro simulated gastric digestion of TiO_2_ NPs representing three different pre-weaning ages resulted in similar levels of aggregation, while no sign of dissolution was observed. These findings are in line with previous published results reported for simulated early life digestion of TiO_2_ E171 [28]. It can therefore be assumed that the administered TiO_2_ NPs display the same physiochemical properties at all three ages tested in vivo in this study.

The intestinal tract is a selective barrier, responsible for nutrient uptake, bidirectional gut-brain interaction, and is host to the gut microbiome. Interference with the homeostasis of the intestinal tract during early life may have long-term health consequences. Metabolomic analysis of plasma collected at PND 21 shows that metabolomic changes are mainly driven by amino acids and glycerophospholipids, suggesting interference with nutrient uptake and metabolism. Furthermore, amino acid biosynthesis and metabolism and aminoacyl-tRNA biosynthesis were dominant among the identified metabolic pathways perturbed due to TiO_2_ NP administration. The metabolomic analysis of plasma demonstrated a significant TiO_2_ NP-induced increase in many individual amino acids, as well as the sum of both non-essential amino acids and total amino acids for all three dosing ages and both sexes. Non-essential amino acids play an important role in a long list of biological functions including regulating gene expression, cell signaling pathways, DNA and protein synthesis, metabolism of glucose and lipids, antioxidative responses, detoxification of xenobiotics and endogenous metabolites, neurotransmission, and immunity [58]. The level of total free amino acids, non-essential, and essential amino acids in plasma has been found to be elevated in obese children with impaired glucose tolerance (age 9–10) [59]. Together, these findings suggest that early life exposure to TiO_2_ NP causes an imbalance in the amino acid profile which could have long-term health impacts e.g., metabolic syndrome. Future investigation is needed to understand and to confirm the mechanism of action behind the TiO_2_ NP induced systemic increase in free amino acids.

The heart undergoes remarkable changes during early life, predominantly at the molecular and metabolic level. Within the first two postnatal weeks the cardiac cells in rodents gradually cease to undergo DNA replication [37, 38], cardiomyocytes cease to proliferate, and the predominant form of growth shifts from hyperplasia to hypertrophy [38, 60]. Soon after birth the changes in metabolic environment induce a shift from anaerobic glycolysis to mitochondrial fatty acid β-oxidation [60, 61]. In this study, no significant changes were found in the cardiac performance of male pups and female pups dosed between PND 2–5, while female pups dosed between PND 7–10 and PND 17–20 had significantly decreased heart rate and increased ST interval. These findings suggest the existence of a sensitive window in female cardiac development or susceptibility between PND 7–20. The metabolomics analysis of endogenous metabolites in plasma offered an insight into possible connections between TiO_2_ NP-induced changes in metabolites and changes in cardiac performance.

Schnackenberg et al., 2016 reported that early stages of cardiotoxicity, induced by the chemotherapeutic agent, doxorubicin, a known cardiotoxin, led to increased plasma levels of acetylornithine and 16 amino acids [62]. In the study presented here, TiO_2_ NP caused an increase in acetylornithine and several amino acids for male and female pups dosed between PND 7–10 and PND 17–20. An increased plasma level of carnitine and several short and long chain acylcarnitines was reported for early stages of doxorubicin induced cardiotoxicity, suggesting an adverse influence on cardiac fatty acid metabolism that might be related to the capacity for β-oxidation of fatty acids [62]. Here we found that several short chain acylcarnitines had an increased FC for male and female pups dosed between PND 7–10, while long chain acylcarnitines had a decreased FC. For pups dosed between PND 17–20 only female pups had a significantly (P-value ≤  0.05) increased FC for short chain acylcarnitines. We speculate that TiO_2_ NP might interfere with the metabolic shift in the heart when administered between PND 7–10 and PND 17–20, with PND 7–10 being the more sensitive window of exposure and female the more sensitive sex. Furthermore, lysophosphatidylcholines (a class of phosphatidylcholines) are among factors that are found to upregulate transcription factors that activate endothelial nitrous oxide synthase (eNOs) production [63]. The primary responsibility of eNOs is the generation of nitrous oxide (NO) which plays a key role in the maintenance of normal blood pressure as well as other important physiological processes such as angiogenesis. NO acts on smooth muscle cells resulting in relaxation of the vessel wall [64, 65]. Therefore, increased production of NO by eNOs activity may be connected to the decrease in the heart rate due to TiO_2_ NP exposure consistent with literature [66, 67]. Interestingly, phosphatidylcholines (glycerophosphocholines) including lysophosphatidylcholines are among the lipids that are found to be important for differentiating TiO_2_ NP dose groups from vehicle controls. Plasma sphingolipids and phospholipids are known to pose risk of cardiovascular disease among other adverse outcomes [68]. In addition, phosphatidylcholines are also considered as a class of metabolites with cardiovascular risk [69, 70]. In our study, these molecules are among the metabolites that were deemed to be important for discrimination of TiO_2_ NP dose groups from control. The changes in ECGs and levels of endogenous metabolites observed in our analysis suggest that there might be a link between early life TiO_2_ NP oral exposure and cardiac performance which need to be further investigated.

The brain undergoes considerable growth and development during early life. Multiple events can impact the biochemistry and homeostasis of the brain including systemic oxidative stress, systemic inflammation, and/or signaling events via the bidirectional gut-brain interaction. Interference with the bidirectional communication between the gut and the brain may lead to complications including inflammatory disorders, obesity, and eating disorders [26, 52]. In rats the blood–brain barrier is established between PND 1–3 [35]. From PND 7–10, the brain undergoes a growth spurt, which coincides with a peak in gliogenesis and an increase in axonal and dendritic density [35]. The pup brain at PND 20–21 has reached 90–95% of its adult weight, however, it experiences a peak in synaptic density and myelination rate at the same time as neurotransmitter and receptor changes [35]. The brain development in rats at PND 1–3 is comparable with pre-term human babies (gestational weeks 23–32), rats at age PND 7–10 comparable with term infants (gestational weeks 36–40), and rats at age PND 20–21 comparable with toddlers age 2–3 years of age [35]. In the study presented here, TiO_2_ NP was found to impact the female pups startle reflex response when administered between PND 2–5 leading to a decreased response in relation to vehicle control. TiO_2_ NP also induce an increase in locomotor activity at PND 20 for female pups dosed between PND 7–10 and a decrease in male pups dosed between PND 17–20. The locomotor activity test is a simple means of assessing spontaneous locomotor activity and arousal in rat pups. Female pups dosed between PND 7–10 showed decrease performance in the rotarod test at PND 20, suggesting that TiO_2_ NP adversely impact the female pups motor coordination and motor skill learning. Interestingly, female pups dosed between PND 7–10 had a significantly increased concentration of DA, while male pups dosed between PND 17–20 had a non-significant decreased DA concentration (P-value = 0.0541). DA levels and locomotor networks have a direct correlation, thus reduced DA concentrations relate to reduced locomotor activity and learning [71, 72], suggesting a possible connection between TiO_2_ NP-induced changes in DA and locomotor performance. The metabolomics analysis offered an insight into possible connections between TiO_2_ NP-induced changes in metabolites and changes in neurobehavioral performance and neurotransmitter levels in the brain. The increased Met-SO/Met ratio measured in both male and female pups dosed between PND 7–10 and PND 17–20 indicates systemic oxidative stress [53], which can impact the biochemistry and homeostasis of the brain, and may negatively impact normal central nervous system functions [73]. The three-tier hierarchical oxidative stress model in nanotoxicology, which is widely accepted, proposes that nanomaterials might present novel mechanisms of injury without introducing new pathology [74]. The hierarchical oxidative stress model describes how: Tier 1, a low level of oxidative stress results in elevation of phase II enzymes and antioxidant enzymes; Tier 2, an intermediate level of oxidative stress results in activation of MAPK and NF-κB cascades induce a pro-inflammatory response; Tier 3, a high level of oxidative stress causes cellular apoptosis and necrosis [74]. For all TiO_2_ NP dose groups there was an increased FC of non-essential amino acids when compared to their vehicle controls. Non-essential amino acids play a central role in neurological function and behavior, through synthesizing neurotransmitters (e.g., DA) [58], serving as agonists or co-agonists at *N*-methyl-d-aspartic acid receptors [58], and conferring neuroprotective reactions [58, 75]. Together these findings suggest that TiO_2_ NP-induced a low level of systemic oxidative stress and changes to non-essential amino acids which result in the observed changes in neurobehavioral performance and neurotransmitter concentrations in brain tissue. How this low level of systemic oxidative stress and changes in non-essential amino acids may induce the neurobiological mechanisms behind the observed sex- and age-related differences in neurobehavioral performance and neurotransmitter concentrations needs further investigation. In recent years, sex differences have been reported in psychopathologies (depression, anxiety, and fear related disorders), which have been suggested to be connected to the corticolimbic system comprising the hippocampus, amygdala, and medial prefrontal cortex [76]. The corticolimbic regions undergo dynamic changes in early life [76] and has been connected with DA neurotransmission and locomotor activity as part of the adaptive system to stress regulation [77]. We speculate that oral TiO_2_ NP administration during early life may interfere with the corticolimbic regions.

While the changes in concentration of neurotransmitters and related metabolites in brain tissue has been reported for oral administration of Al_2_O_3_ NP between PND 17–20 in male and female rat pups [26], it has not previously been investigated if oral exposure to TiO_2_ NPs in pre-weaning rats interfere with the biochemistry of the brain and influence neurobehavior. Collectively, these data suggest that TiO_2_ NP can impact behavior and change the concentrations of neurotransmitters and related metabolites in the brain of rat pups following oral exposure, and that both age of exposure and sex play a role. More research is needed to understand the underlying mechanisms behind these effects and the long-term consequences of these TiO_2_ NP induced interferences with neurobehavior and brain biochemistry and the connection with changes in endogenous metabolite profiles.

## Conclusion

The developmental origins of health and disease (DOHaD) paradigm states that the origin of many diseases takes place during development, even if the clinical manifestation happens throughout the lifespan [78–80]. Altered nutrition or exposure to environmental chemicals and toxins during development may have persistent, adverse effects with long-term health consequences. This suggests that maximizing healthy growth and minimizing injury during in utero and early childhood development is critical to attaining optimal function in adulthood, which is the primary protection against development of diseases and dysfunctions. Results presented here demonstrate that early life exposure to TiO_2_ NPs has adverse effects on cardiac performance and neurobehavioral assessment, causes perturbations in brain biochemistry and plasma metabolite profiles, and age and sex appear to play an important role in the biological outcome. It is therefore important to further investigate the potential short- and long-term health impacts of ingested TiO_2_ NP during early life; if it adversely influences cardiovascular and cognitive development, liver functions, and possibly contribute to metabolic diseases and dysfunctions across the lifespan.

## Methods

### Nanomaterials and chemicals

The TiO_2_ NP (TiO_2_ P25) used in the studied reported here was procured, comprehensively characterized, and provided to us by the Engineered Nanomaterials Resource and Coordination Core (ERCC), as part of National Institute of Environmental Health Sciences (NIEHS) Nanotechnology Health Implications Research (NHIR) Consortium. Solvents and chemicals for in vitro digestion studies included sodium chloride (NaCl), sodium phosphate monobasic (NaH_2_PO_4_), potassium chloride (KCl), calcium chloride dihydrate (CaCl_2_(H_2_O)_2_), ammonium chloride (NH_4_Cl), hydrochloric acid (HCl), glucose (C_6_H_12_O_6_), glucuronic acid (C_6_H_10_O_7_), glucosamine hydrochloride, pepsin, sodium bicarbonate (NaHCO_3_), monobasic potassium phosphate (KH_2_PO_4_), and magnesium chloride (MgCl_2_), and were procured from Fisher Scientific (Suwanee, GA). Urea, bovine serum albumin (BSA), mucin, pancreatin, lipase, and bile were purchased from Sigma-Aldrich (St. Louis, MO). Pepsin from porcine gastric mucosa was purchased from Thermo Fisher Scientific (Waltham, MA). Nitric acid for Inductively Coupled Plasma Optical Emission Spectrometry (ICP-OES) analysis was purchased from Fisher Scientific (Suwanee, GA). For neurotransmitter analysis, citric acid, the internal standard 3,4-dihydroxybenzylamine (DHBA), sodium phosphate (monobasic) NaH_2_PO_4_, EDTA, octanesulfonic acid, dopamine (DA), dihydroxyphenylacetic acid (DOPAC), homovanillic acid (HVA), 5-hydroxyindole-3-acetic acid (5-HIAA), norepinephrine (NE), and serotonin (5-HT) were purchased from Sigma-Aldrich (St. Louis, MO).

### In vitro gastric digestion studies

In vitro studies of simulated gastric digestion were conducted for pre-weaning rat gastric juice simulating three different ages and gastric phases: bland phase (~ PND 7), transitional phase (~ PND 14), and acidic phase (~ PND 21) for 1, 2, and 4 h (Additional file 6: Table S4) as previously described in the literature [28]. In short, the formulation of the acidic phase, resembling that of the adult rat, was performed as published by Chen et al., 2013 [81]. At birth and up until PND 7 the pH of the rat stomach was neutral, but at PND 14 it dropped to pH 6, and continued to drop until it reached pH 4 at PND 21 [40]. Up to PND 7, there was no gastric HCl production [40]. At PND 14, pepsin production had started [40]. In the study presented here, the transitional gastric phase was mimicked by using half of the adult pepsin level in the transitional phase. At PND 7, no mucus producing cells are present [40], so mucin was only included in the transitional and acidic phase.

The simulated gastric digestion was conducted at 37 °C at a NP concentration of 0.25 mg/mL. Multiple time points (1, 2, and 4 h) were tested for each gastrointestinal solution reflecting a range of possible gastrointestinal transit times. Digested NPs were characterized by dynamic light scattering (DLS), scanning electron microscope (SEM), and Inductively Coupled Plasma Optical Emission Spectrometry (ICP-OES) analysis, as described below.

### Nanoparticle formulation and stability test

TiO_2_ NP dosing solution (2 mg/mL) was formulated and freshly prepared every day for in vivo exposure studies. TiO_2_ NP was formulated in filtered deionized water and sonicated in a cup horn sonicator (Ultrasonic Liquid Processor S-4000, Misonix Inc., Farmingdale, NY) following the published ‘Discrete Sonication’ protocol by Cohen et al., 2018 [82] which was slightly modified as previously described [26, 28]. The critical delivered sonication energy (DSEcr, J/mL) was determined by measuring the hydrodynamic diameter using dynamic light scattering (DLS, Malvern Zetasizer Nano-ZS, Malvern Panalytical, Westborough, MA) every 2–5 min, until the change in NP diameter was less than 5%. DSEcr for the TiO_2_ NP dosing solution was determined to be 2,930 J/mL. The dispersion stability of the TiO_2_ NP dosing formulation was measured in water after 0 h and 4 h by DLS, to ensure stability between formulation and completion of dosing.

### Nanoparticle characterization

The size, uniformity, and morphology of TiO_2_ NP were characterized using a transmission electron microscope (TEM) (LIBRA®120, Carl Zeiss Microscopy, Peabody, MA). For TEM, imaging samples were prepared by placing a drop of NP suspension at low density on formvar-coated TEM grids (Ted Pella, Redding, CA), followed by rinsing in DDH_2_O and finally, drying at room temperature.

Digested TiO_2_ NP were characterized using DLS (Malvern Zetasizer Nano-ZS, Malvern Panalytical, Westborough, MA), SEM, and ICP-OES. SEM was performed using a Zeiss Auriga field emission scanning electron microscope (FESEM) (Carl Zeiss Microscopy, White Plains, NY) at 5 kV accelerating voltage and a beam current of 10 µA. Prior to ICP-OES analysis each sample of 5 mL TiO_2_ NP in gastric fluid was filtered through a 5000 molecular weight cut off centrifuge filter (Sartorius Vivaspin, Goettingen, Germany) (cut off < 2 nm) at 8000 rpm for 20 min, to filter out undigested NPs. Overnight digested of filtrate and filtered NPs were done in 3% nitric acid overnight and analyzed by ICP-OES. Limit of detection (LOD) was 50 ng Ti mL-1. LOC was calculated in terms of three times the standard deviation (98% confidence) of 10 replicates of blank measures in accordance with the recommendation of International Union of Pure and Applied Chemistry (IUPAC).

TiO_2_ NP dose solutions characterized using DLS (Malvern Zetasizer Nano-ZS, Malvern Panalytical, Westborough, MA), and nanoparticle tracker (NTA) (NanoSight LM10, Malvern Panalytical, Westborough, MA).

### Housing and dose administration

Sprague Dawley rats were obtained from Charles River Laboratories (Raleigh, NC). Pregnant Sprague Dawley rats arrived on gestational day (GD) 16–17 (for groups dosed between PND 2–5). Litters were standardized prior to dosing at PND 2. Lactating dams with their standardized litter of 5 male and 5 female pups arrived on PND 2–3 or PND 11–12 (for dosing groups dosed between PND 7–10 or PND 17–20, respectively). Each dosing group contained three litters, equal to a total of 15 female and 15 male pups. The animals were handled, cared for, and used in compliance with the Guide for the Care and Use of Laboratory Animals [83] and approved by the Institutional Animal Care and Use Committee (IACUC) of Mispro Biotech, Research Triangle Park, NC, USA. Dams were housed with their litter in individual polycarbonate cages and fed LabDiet 5058 Breeder Diet (LabDiet, Durham, NC) and Durham City (NC) water from a reverse osmosis system provided ad libitum. The animal room was maintained at 72 ± 3 °F, 30–70% relative humidity and a 12:12 light cycle. All rats were acclimated 5–7 days prior to initiation of dosing.

Pups received a daily oral dose of 10 mg/kg bw/day TiO_2_ NP between PND 2–5, PND 7–10, or PND 17–20. Dosing was conducted at the same time each day for all dosing groups. The vehicle control was administered an equal volume of deionized water. Each pup was weighed on each dosing day and the appropriate volume of the dosing solution (based on body weight) administered. Pups aged PND 2–5 were dosed via a suckling procedure, such that the dose was slowly administered via a pipette in the corner of the mouth so that the pup suckled and swallowed the dose. Pups aged PND 7–20 were dosed via a full gavage procedure, where a stainless steel 22G ball-tipped gavage dosing needle was fully inserted into the esophagus.

The pups were euthanized by live decapitation at PND 21, since using CO_2_ or anesthetic agents can cause dramatic changes in neurotransmitters [84, 85]. Trunk blood was collected right after decapitation and processed to plasma. The liver and brain were collected and weighed. The brain was sectioned longitudinally and placed in separate containers; the right-brain halves were used in the analysis to determine the concentration of six neurotransmitters and related metabolites as described below.

### Cardiac assessment

ECGenie (Mouse Specific, Inc, Framingham, MA) was utilized to measure cardiac repolarization (electrocardiograms [ECG]) on rat pups at PND 20. The pups were placed on the platform for 10 min to allow them to acclimate and to record good quality ECGs. A minimum of five good quality ECG sequences were recorded for each pup and the mean echocardiographic parameters were calculated. For pups dosed PND 17–20, the cardiac repolarization was measured 4 h after the last dose was administered. The cardiac assessment was carried out for six to seven pups of each sex from three litters and conducted at the same time for male and female pups. The ECGenie detects cardiac electrical activity through the animals’ paws, using a shielded acquisition platform, analog input and bioamplification, and direct connection to a computer with the data acquisition software (LabChart8).

### Basic neurobehavioral assessment

Basic neurobehavior assessments were conducted on PND 20 for a total of eight male and eight female pups chosen randomly from three litters using acoustic startle response test, locomotor activity test, and rotarod test as previously described [26]. For the dosing groups that received TiO_2_ NPs between PND 17–20 and aged match vehicle control, the assessment was performed at 4 h after administration of the last dose. Testing was conducted at the same time for male and female pups.

The Startle Response System Test (San Diego Instruments SR-Lab chambers, San Diego, CA) was utilized for acoustic startle response testing. The session started after a 5-min (300 s) acclimation period where pups were exposed to background noise of 69 dB, followed by the acoustic startle as a single pulse of 120 dB. Acoustic startle was measured as time to max peak (msec), Average Peak (mV), and Max Peak (mV).

The locomotor activity test was done using the Photobeam Activity System (PAS) (San Diego Instruments, San Diego, CA). The photobeam test is designed to assess the locomotor activity using the collection and recording of beam breaks over time as the animal moves. Locomotor activity was recorded for 10 min in two intervals; Interval 1: 0:00–5:00 min and Interval 2: 5:01–10:00 min. Locomotor activity was examined in two 5-min intervals to examine any potential changes in locomotor activity as a result of TiO_2_ NP administration.

The Rotarod test was performed using a Stoelting Rotarod, model 52,790, using 1¼ inch diameter drums (Stoelting, Wood Dale, IL), and measured as time to fall (sec) and distance to fall. The test was completed when all pups fell off the rod, or when 150 s had expired, and the rod stopped moving.

### Neurotransmitter and related metabolite analysis of brain tissue

Quantification of six monoamine neurotransmitters and related metabolites, dopamine (DA), dihydroxyphenylacetic acid (DOPAC), homovanillic acid (HVA), norepinephrine (NE) (also called noradrenaline (NA) or noradrenalin), serotonin (5-HT), and 5-hydroxyindole-3-acetic acid (5-HIAA), which are related to memory, emotion, depression, anxiety, and neuroendocrine function, was done in the right brain half from eight male and eight female pups using ultra high pressure liquid chromatography (UPLC) coupled with electrochemical detection (ECD) as previously described [26]. In short, brain tissue was prepared for analysis in tissue buffer (0.05 M Na_2_HPO_4_, 0.03 M citric acid, and 2 mM ascorbic acid at pH 3). Internal standard solution was prepared with 3,4-dihydroxybenzylamine (DHBA) at 200 ng/mL in tissue buffer. Internal standard solution was then added to each sample at a ratio of 5 mL per g of brain tissue. Neurotransmitter and related metabolite extraction were conducted by homogenizing the brain tissue using ten, 2.8 mm stainless steel grinding balls (OPS Diagnostics, Lebanon, NJ) in a Geno/Grinder 2010 (SPEX SamplePrep, Metuchen, NJ). After homogenization, the samples were centrifuged at 3500×*g* for 10 min at 4 °C. Aliquots of supernatant were taken and passed through an Ultrafree®-MC 0.45 μm Polyvinylidene Fluoride (PVDF) filter (Merck Millipore Ltd., Tullagreen Carrigtwohill, Co. Cork, IRL). The processed samples were analyzed by injecting a 10-µL aliquot onto a Luna Omega 1.6 μm Polar C18, 2.1 × 150 mm column (Phenomenex, Torrance, CA) coupled to an LPG-3400RS pump, WPS-3000TBRS autosampler, and a 5600A CoulArray electrochemical detector (Thermo Scientific, Waltham, MA). The column was heated to 32 °C. The mobile phase consisted of 50 mM sodium phosphate, 47 mM citric acid, 0.14 mM EDTA, 0.64 mM octanesulfonic acid, and 5% methanol, and was delivered isocratically at a flow rate of 0.4 mL/min. The detector was set to sequentially deliver potentials of -150 mV, 150 mV, 400 mV, and 600 mV.

### Metabolomics analysis

Plasma samples from 14–15 males and 14–15 females per dosing group and reference plasma were processed for targeted metabolomics analysis by liquid chromatography-mass spectrometry (LC–MS/MS) using the AbsoluteIDQ p180 kit (Biocrates Life Sciences AG, Innsbruck, Austria). The kit quantifies 40 acylcarnitines, 21 amino acids, 1 monosaccharide, 90 glycerophospholipids, 15 sphingolipids, and 21 biogenic amines, in addition calculates 44 metabolite ratios. Samples were processed as described in the AbsoluteIDQ Kit user manual p180 (Biocrates Life Sciences AG, Innsbruck, Austria). Aliquots (10 µL) of plasma samples were used in the analysis according to the AbsoluteIDQ Kit instructions [86, 87]. Reference plasma comprised pooled plasma from dams, four reference plasma samples were included per plate. LC–MS/MS analysis and flow injection were conducted on an API 4000 triple quad mass spectrometer (AB Sciex, Framingham, MA) coupled with an Agilent 1100 high performance liquid chromatography (HPLC) (Agilent Technologies, Palo Alto, CA) as described in the AbsoluteIDQ Kit user manual. All data was processed using Analyst 1.6.2 (AB Sciex, Framingham, MA) and MetIDQ Nitrogen 7 software (Biocrates Life Sciences AG, Innsbruck, Austria). Values below limit of detection (LOD) were not included in the statistical analysis.

### Statistical and multivariate analysis

A nonparametric Mann–Whitney U-test was performed for organ-bw-ratio, cardiac assessment, neurobehavior assessment, and neurotransmitter and related metabolite concentration data to test for statistical differences between dosing groups using the software GraphPad Prism 7.04 (GraphPad Software, San Diego, CA).

The univariate statistical analysis for plasma metabolite data was conducted using Biocrates MetIDQ Nitrogen 7 with the Biocrates MetIDQ StatPack (Biocrates, Life Sciences AG, Innsbruck, Austria). The statistical significance of metabolites between the dosing groups and their corresponding vehicle controls was evaluated using a Mann–Whitney U-test. The metabolite fold-change (FC) between individual metabolites were calculated using the median. Nominal P-values are reported for the comparison between TiO_2_ NP treated and the vehicle controls.

Multivariate analysis of the metabolomics data was performed, using SIMCA 15.0 (Sartorius Stedim Data Analytics, AB, Umeå, Sweden) to reduce the dimensionality and to enable the visualization of the differentiation of the dosing groups [88, 89]. Data were mean-centered and unit variance (UV) scaled prior to multivariate data analysis. Unsupervised models were created using principal component analysis (PCA) and the scores plots were inspected to ensure that the quality control (QC) pool samples were tightly clustered, and in the center of the study samples from which they were derived—a QC method that is widely used in metabolomic studies [90]. Supervised analysis, orthogonal partial least squares discriminate analysis (OPLS-DA), was used to determine the metabolites deemed important for differentiating the study groups based on variable influence on projection (VIP) scores. VIP ≥ 1.0 with a jack-knife confidence interval that did not include 0 was considered as important. The VIP statistic summarizes the importance of the metabolites in differentiating the dosing groups [88]. All models used a sevenfold cross-validation to assess the predictive ability of the model (Q2).

Metabolites that had VIP ≥ 1.0 and/or P-value ≤ 0.05 were deemed to be important for differentiating the dosing groups against their respective controls.

### Metabolic pathway analysis

MetaboAnalyst software [91] was used for pathway analysis to assess the perturbated metabolic pathways. Metabolites deemed to be important in the multivariate and univariate statistical data analysis (P-value ≤ 0.05 or VIP ≥ 1.0 were selected as important) were used as input for MetaboAnalyst. For pathway analysis, the Over-Representation Analysis (ORA) method hypergeometric test was selected and the pathway library for rats (KEGG code rno; Rattus norvegicus) was used. Relative betweenness centrality was selected as node importance measure for pathway topological analysis. The Metabolite Set Enrichment Analysis (MSEA) was used to evaluate consistent changes among lipids deemed to be important, using the ORA method hypergeometric test and the “Sub-class” metabolite set library that contains 1072 sub chemical class metabolite sets or lipid sets (based on the chemical structures). A false discovery rate (FDR) correct P-value ≤ 0.05 is indicative of significant perturbation in a pathway.

## Supplementary Information

**Additional file 1: Figure S1.** SEM images of pristine TiO2 NP (A) and in vitro digestion of TiO2 NP in gastric fluids simulating (B) bland phase (~PND 7, pH = 7), (C) transitional phase (~PND 14, pH = 6), and (D) acidic phase (PND 21, pH = 4)

**Additional file 2: Figure S2.** Pie chart showing the six metabolite classes (Amino Acids, Biogenic Amines, Sugars, Acylcarnitines, Glycerophospholipids, and Sphingolipids) with a VIP > 0.95 with an S.E. less than mean (A, C, E, G, I, K) and significant P-value <0.05 (Mann-Whitney U test) (B, D, F, H, J, L). Rat pups were dosed between PND 2–5 (A–D), PND 7–10 (E–H), or PND 17–20 (I–L) and sacrificed on PND 21. The total number of metabolites deemed to be important for differentiating the dosing groups against their respective controls is listed under each pie-chart. Amino acids were the predominant metabolite class with high VIPs and therefore drove the differentiation of dosing groups in the OPLS-DA. Glycerophospholipids were the predominant metabolite class for significantly different metabolites between TiO2 NP and vehicle control. N = 15

**Additional file 3: Table S1.** In vitro gastric digestion of TiO_2_ NP in gastric fluids of rat pups, bland phase (~PND 7, pH = 7), transitional phase (~PND 14, pH = 6), and acidic phase (PND 21, pH = 4). The hydrodynamic diameter of TiO_2_ NP in dH_2_O was 440 ± 68.2 nm, and incubation of TiO_2_ NP in all three phases led to aggregation and an increase hydrodynamic diameter. ICP-OES showed no dissolution of TiO_2_ NP; limit of detection (LOD) for Ti was 50 μg/L.

**Additional file 4: Table S2.** Metabolites in plasma collected from male pups (n = 14–15) with significant P-value ≤ 0.05 and/or VIP > 1.0 with an S.E. less than mean. Metabolites with a P-value ≤ 0.1 are also shown.

**Additional file 5: Table S3.** Metabolites in plasma collected from female pups (n = 14–15) with significant P-value ≤ 0.05 and/or VIP > 1.0 with an S.E. less than mean. Metabolites with a P-value ≤ 0.1 are also shown.

**Additional file 6: Table S4.** Chemical formulation of digestion of in *vitro rat* gastrointestinal solutions tested in this study and time (h) of digestions.

## Data Availability

Data is contained within the article or supplement material.

## Abbreviations

5-HIAA: 5-hydroxyindole-3-acetic acid
5-HT: Serotonin
Ag NPs: Silver nanoparticles
Al_2_O_3_ NPs: Aluminum oxide nanoparticles
bw: Body weight
CuO NPs: Copper oxide nanoparticles
DA: Dopamine
DLS: Dynamic light scattering
DOPAC: Dihydroxyphenylacetic acid
ECG: Electrocardiogram
eNOS: Endothelial nitrous oxide synthase
FC: Fold change
FDR: False discovery rate
HPLC: High performance liquid chromatography
HVA: Homovanillic acid
ICP-OES: Inductively coupled plasma-optical emission spectrometry
LC–MS/MS: Liquid chromatography–mass spectrometry
LOD: Level of detection
LPC: Lysophosphatidylcholines
LysoPC: Lysophosphatidylcholines
Met-SO: Methionine sulfoxide
MSEA: Metabolite set enrichment analysis
NE: Norepinephrine
NIEHS: National Institute of Environmental Health Sciences
NHIR: Nanomaterials Health Implications Research
NO: Nitrous oxide
NPs: Nanoparticles
NTA: Nanoparticle tracking analysis
OPLS-DA: Orthogonal partial least squares discriminate analysis
ORA: Over-representation analysis
PC: Glycerophospholipids
PCA: Principal component analysis
PdI: Polydispersity index
PND: Postnatal day
QC: Quality control
SEM: Scanning electron microscope
SM: Sphingomyelins
SM (OH): Hydroxylated sphingomyelins
TEM: Transmission electron microscopy
TiO_2_: Titanium dioxide
UV: Unit variance
VIP: Variable influence on projection
vs: Versus
