# Supplementary figures and images for: Oral administration of TiO2 nanoparticles during early life impacts cardiac and neurobehavioral performance and metabolite profile in an age- and sex-related manner
Source: Part Fibre Toxicol. 2022 Jan 5;19:3. doi: 10.1186/s12989-021-00444-9 (PMC8728993; doi:10.1186/s12989-021-00444-9)

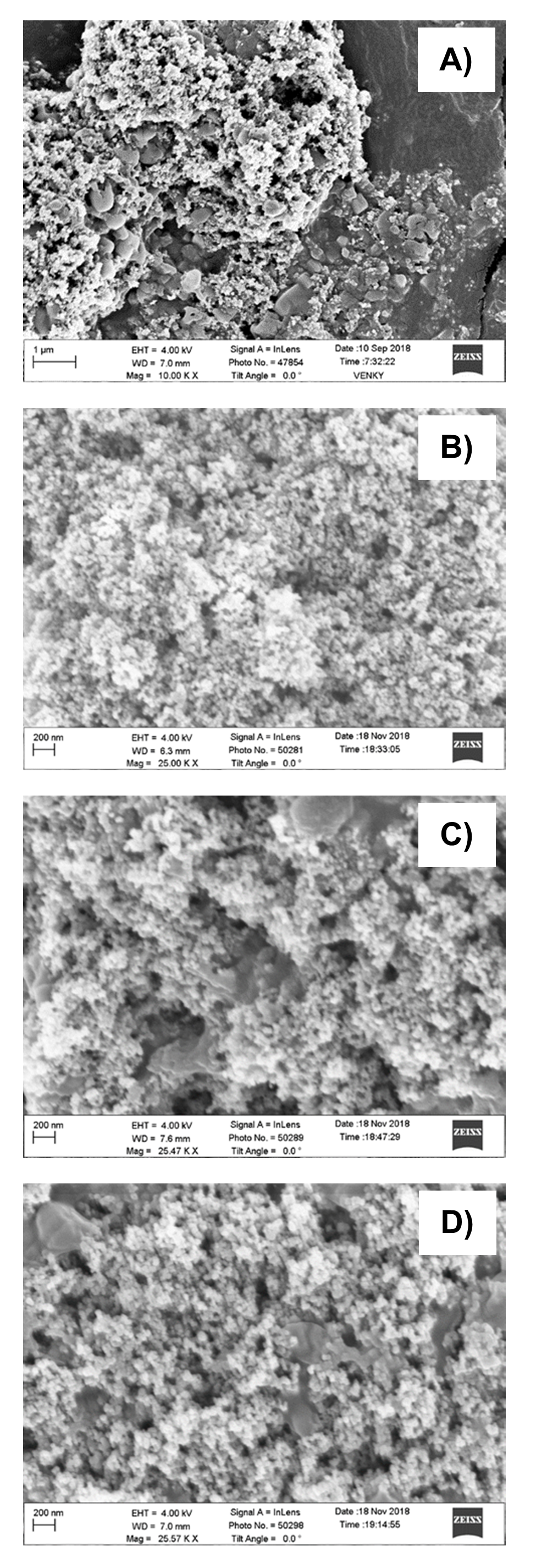

Supplement: Supplementary file 1 — Additional file 1: Figure S1. SEM images of pristine TiO2 NP (A) and in vitro digestion of TiO2 NP in gastric fluids simulating (B) bland phase (~PND 7, pH = 7), (C) transitional phase (~PND 14, pH = 6), and (D) acidic phase (PND 21, pH = 4) [file 12989_2021_444_MOESM1_ESM.tif]

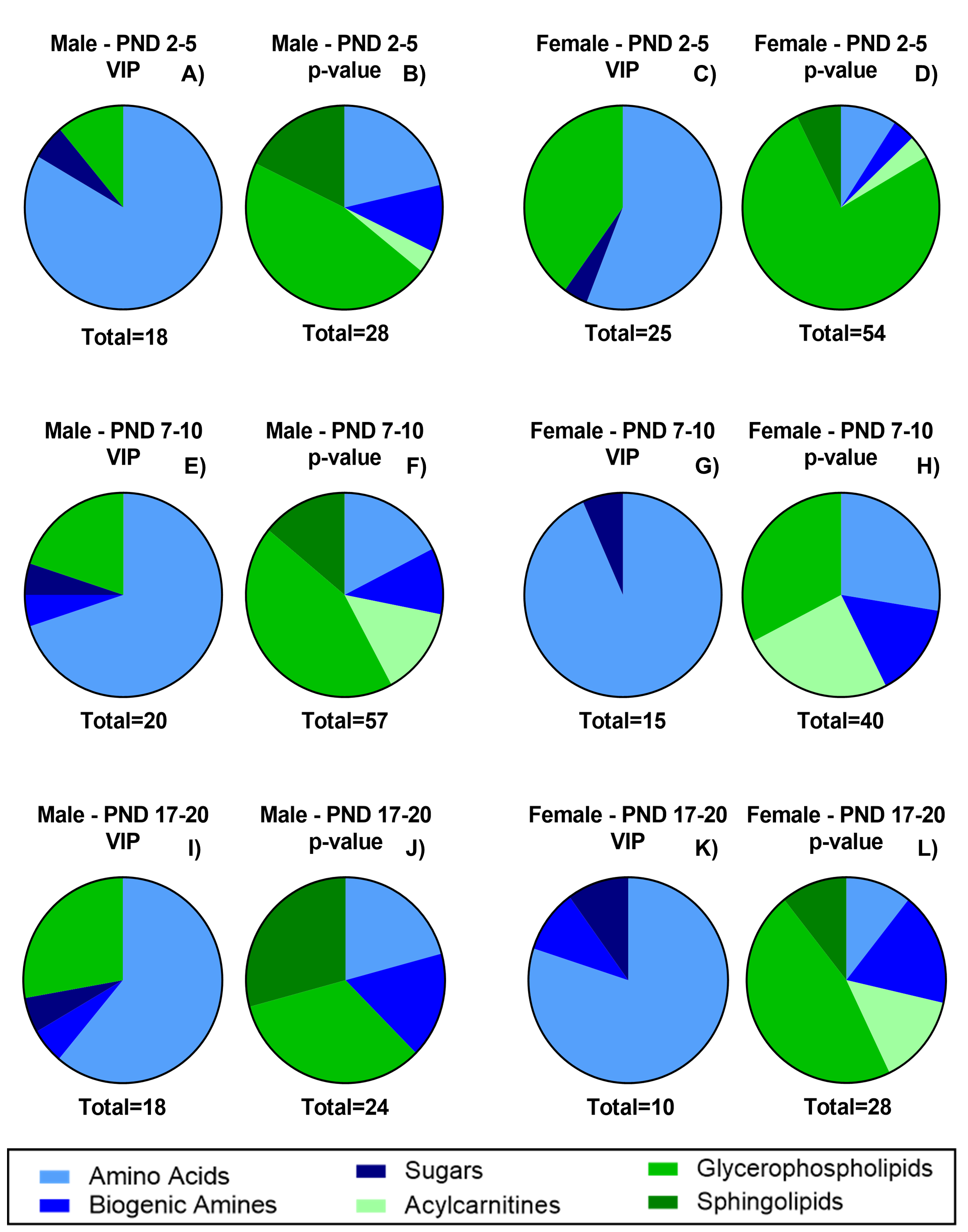

Supplement: Supplementary file 2 — Additional file 2: Figure S2. Pie chart showing the six metabolite classes (Amino Acids, Biogenic Amines, Sugars, Acylcarnitines, Glycerophospholipids, and Sphingolipids) with a VIP > 0.95 with an S.E. less than mean (A, C, E, G, I, K) and significant P-value <0.05 (Mann-Whitney U test) (B, D, F, H, J, L). Rat pups were dosed between PND 2–5 (A–D), PND 7–10 (E–H), or PND 17–20 (I–L) and sacrificed on PND 21. The total number of metabolites deemed to be important for differentiating the dosing groups against their respective controls is listed under each pie-chart. Amino acids were the predominant metabolite class with high VIPs and therefore drove the differentiation of dosing groups in the OPLS-DA. Glycerophospholipids were the predominant metabolite class for significantly different metabolites between TiO2 NP and vehicle control. N = 15 [file 12989_2021_444_MOESM2_ESM.tif]
