# Supplementary material for: Oral administration of TiO2 nanoparticles during early life impacts cardiac and neurobehavioral performance and metabolite profile in an age- and sex-related manner
Source: Part Fibre Toxicol. 2022 Jan 5;19:3. doi: 10.1186/s12989-021-00444-9 (PMC8728993; doi:10.1186/s12989-021-00444-9)
Supplement: Supplementary file 3 — Additional file 3: Table S1. In vitro gastric digestion of TiO2 NP in gastric fluids of rat pups, bland phase (~PND 7, pH = 7), transitional phase (~PND 14, pH = 6), and acidic phase (PND 21, pH = 4). The hydrodynamic diameter of TiO2 NP in dH2O was 440 ± 68.2 nm, and incubation of TiO2 NP in all three phases led to aggregation and an increase hydrodynamic diameter. ICP-OES showed no dissolution of TiO2 NP; limit of detection (LOD) for Ti was 50 μg/L. [file 12989_2021_444_MOESM3_ESM.docx]

**Supplement Table 1.** *In vitro* gastric digestion of TiO_2_ NP in gastric fluids of rat pups, bland phase (~PND 7, pH = 7), transitional phase (~PND 14, pH = 6), and acidic phase (PND 21, pH = 4). The hydrodynamic diameter of TiO_2_ NP in dH_2_O was 343 ± 8.9 nm, and incubation of TiO_2_ NP in all three phases led to aggregation and an increase hydrodynamic diameter. ICP-OES showed no dissolution of TiO_2_ NP; limit of detection (LOD) for Ti was 50 μg/L.

| **Gastric Phase (Age)** | **Inc. time** | **DLS** | **ICP-OES** |
| --- | --- | --- | --- |
|  |  | **TiO_2_ NP**  **Diameter [nm], average ± standard deviation; (PdI)** | **TiO_2_ NP**  **%Ti digested in filtrate (compared to initial Ti)** |
| **Control** | 0 h | 343 ± 8.9 (0.23) | <LOD |
| **Bland Phase**  **(PND 7)** | 1 h | 1,572 ± 216 (0.864) | <LOD |
|  | 2 h | 1,407 ± 307 (1.00) | <LOD |
|  | 4 h | 825 ± 110 (0.528) | <LOD |
| **Transitional Phase (PND 14)** | 1 h | 1,494 ± 253 (0.743) | <LOD |
|  | 2 h | 1,659 ± 254 (0.969) | <LOD |
|  | 4 h | 1,790 ± 98.5 (0.767) | <LOD |
| **Acidic Phase**  **(PND 21)** | 1 h | 1,052 ± 132 (0.595) | <LOD |
|  | 2 h | 936 ± 95.1 (0.651) | <LOD |
|  | 4 h | 941 ± 76.3 (0.578) | <LOD |
