# Supplementary material for: Oral administration of TiO2 nanoparticles during early life impacts cardiac and neurobehavioral performance and metabolite profile in an age- and sex-related manner
Source: Part Fibre Toxicol. 2022 Jan 5;19:3. doi: 10.1186/s12989-021-00444-9 (PMC8728993; doi:10.1186/s12989-021-00444-9)
Supplement: Supplementary file 4 — Additional file 4: Table S2. Metabolites in plasma collected from male pups (n = 14–15) with significant P-value ≤ 0.05 and/or VIP > 1.0 with an S.E. less than mean. Metabolites with a P-value ≤ 0.1 are also shown. [file 12989_2021_444_MOESM4_ESM.docx]

**Supplement Table 2.** Metabolites in plasma collected from male pups (n = 14–15) with significant P-value ≤ 0.05 and/or VIP > 1.0 with an S.E. less than mean. Metabolites with a P-value ≤ 0.1 are also shown.

|  | **PND 2–5** | | | **PND 7–10** | | | **PND 17–20** | | |
| --- | --- | --- | --- | --- | --- | --- | --- | --- | --- |
| **Metabolite** | **p-value** | **VIP** | **fold change** | **p-value** | **VIP** | **fold change** | **p-value** | **VIP** | **fold change** |
| **Amino Acids** |  |  |  |  |  |  |  |  |  |
| Alanine | 0.0779 | 3.64 | 1.09 | 0.000125 | 5.49 | 1.17 | 0.0037 | 5.24 | 1.16 |
| Arginine | 0.00113 | 3.52 | 1.22 | - | - | - | 0.00883 | 3.89 | 1.43 |
| Asparagine | - | - | - | 0.0171 | 1.68 | 1.12 | - | - | - |
| Aspartate | - | - | - | 0.0107 | 1.08 | 0.721 | - | - | - |
| Citrulline | - | - | - | 0.00746 | 2.89 | 1.24 | 0.000034 | 4.43 | 1.30 |
| Glutamine | 0.00323 | 6.40 | 1.18 | 0.0279 | 4.83 | 1.22 | 0.0771 | 3.76 | 1.07 |
| Glutamate | 0.130 | 0.990 | 1.04 | - | - | - | 0.0606 | 0.64 | 1.12 |
| Glycine | 0.0327 | 3.17 | 1.05 | 0.290 | 2.71 | 0.968 | - | - | - |
| Histidine | - | - | - | - | - | - | 0.256 | 1.01 | 1.05 |
| Isoleucine | 0.648 | 1.01 | 0.984 | - | - | - | 0.0809 | 1.327 | 0.931 |
| Leucine | 0.130 | 2.12 | 1.01 | 0.272 | 1.47 | 1.02 | 0.570 | 1.15 | 0.950 |
| Lysine | 0.917 | 3.22 | 0.985 | 0.000173 | 2.41 | 1.32 | 0.00345 | 4.88 | 0.692 |
| Methionine | 0.135 | 1.57 | 1.11 | - | - | - | 0.00260 | 1.78 | 1.25 |
| Ornithine | 0.0400 | 1.61 | 1.14 | 0.0930 | 1.20 | 1.11 | 0.0928 | 1.69 | 0.838 |
| Phenylalanine | - | - | - | 0.00952 | 1.51 | 1.20 | - | - | - |
| Proline | 0.171 | 1.93 | 1.09 | 0.00246 | 3.12 | 1.18 | 0.0809 | 2.05 | 1.08 |
| Serine | 0.663 | 1.28 | 1.17 | 0.0488 | 2.72 | 1.17 | 0.600 | 1.70 | 1.06 |
| Threonine | 0.120 | 2.05 | 1.10 | 0.0564 | 1.69 | 1.19 | 0.0845 | 1.697 | 0.902 |
| Tryptophan | 0.0442 | 1.42 | 1.19 | - | - | - | - | - | - |
| Tyrosine | - | - | - | 0.00745 | 1.83 | 1.15 | - | - | - |
| Valine | 0.00659 | 2.78 | 1.07 | 0.0852 | 1.66 | 1.09 | - | - | - |
| **Biogenic Amines** |  |  |  |  |  |  |  |  |  |
| Acetylornithine | - | - | - | 0.00001 | 0.933 | 1.82 | 0.000345 | 0.732 | 1.66 |
| Asymmetric dimethylarginine | 0.0488 | 0.296 | 1.30 | - | - | - | 0.00167 | 0.273 | 1.59 |
| Carnosine | 0.0712 | 0.385 | 1.36 | - | - | - | - | - | - |
| Histamine | 0.0876 | 0.105 | 1.26 | - | - | - | - | - | - |
| Methionine sulfoxide (Met-SO) | - | - | - | **0.000081** | 0.761 | **1.49** | **0.000071** | 0.859 | **1.62** |
| Putrescine | 0.0464 | 0.168 | 1.16 | 0.0712 | 0.110 | 0.896 | - | - | - |
| Serotonin | 0.00264 | 0.507 | 1.17 | - | - | - | - | - | - |
| Spermidine | - | - | - | 0.00953 | 0.179 | 0.869 | - | - | - |
| Spermine | - | - | - | 0.0136 | 0.113 | 0.774 | - | - | - |
| Trans-4-Hydroxyproline (t4-OH-Pro) | - | - | - | 0.0488 | 0.919 | 0.815 | 0.0447 | 0.918 | 1.14 |
| Taurine | 0.0971 | 0.778 | 1.08 | 0.0153 | 2.16 | 0.869 | 0.727 | 1.55 | 1.06 |
| **Sugar** |  |  |  |  |  |  |  |  |  |
| Hexoses (including glucose) | 0.272 | 7.35 | 1.06 | 0.576 | 6.28 | 0.99 | 0.861 | 6.00 | 1.02 |
| **Acylcarnitines** |  |  |  |  |  |  |  |  |  |
| Propionylcarnitine | - | - | - | **0.000075** | 0.153 | **1.34** | 0.0668 | 0.063 | 1.04 |
| Malonylcarnitine (Hydroxybutyrylcarnitine) | - | - | - | 0.00871 | 0.0463 | 1.19 | - | - | - |
| Butyrylcarnitine | 0.0107 | 0.0775 | 1.28 | - | - | - | 0.0731 | 0.115 | 1.14 |
| Valerylcarnitine | - | - | - | 0.0250 | 0.0262 | 1.37 | - | - | - |
| Hydroxyvalerylcarnitine (Methylmalonylcarnitine) | - | - | - | 0.0233 | 0.0390 | 1.33 | - | - | - |
| Tetradecenoylcarnitine | - | - | - | 0.0367 | 0.0322 | 0.907 | - | - | - |
| Hexadecanoylcarnitine | - | - | - | 0.00230 | 0.0511 | 0.855 | - | - | - |
| Octadecanoylcarnitine | - | - | - | 0.0152 | 0.0343 | 0.829 | - | - | - |
| Octadecadienylcarnitine | - | - | - | 0.0205 | 0.0245 | 0.806 | - | - | - |
| **Glycerophospholipids (Lysophosphatidylcholines [lysoPC]; Phosphatidylcholines [PC])** | | | | | | | | | |
| lysoPC a C16:1 | 0.0362 | 1.15 | 1.05 | - | - | - | 0.0362 | 0.154 | 1.09 |
| lysoPC a C17:0 | 0.0213 | 0.225 | 1.22 | - | - | - | 0.00124 | 0.220 | 1.13 |
| lysoPC a C18:0 | - | - | - | - | - | - | 0.0736 | 0.867 | 1.09 |
| lysoPC a C18:1 | - | - | - | 0.0265 | 0.507 | 0.884 | - | - | - |
| lysoPC a C20:4 | - | - | - | 0.0443 | 0.634 | 0.951 | - | - | - |
| lysoPC a C28:0 | - | - | - | 0.0328 | 0.0672 | 1.33 | 0.0167 | 0.052 | 1.26 |
| PC aa C32:0 | - | - | - | 0.0265 | 0.392 | 0.906 | 0.0325 | 0.567 | 0.933 |
| PC aa C32:1 | 0.0488 | 0.261 | 1.08 | 0.0852 | 0.269 | 0.904 | - | - | - |
| PC aa C32:2 | 0.0712 | 0.267 | 1.182 | 0.0649 | 0.305 | 1.36 | - | - | - |
| PC aa C34:1 | - | - | - | 0.00747 | 0.964 | 0.869 | - | - | - |
| PC aa C34:2 | 0.0107 | 0.811 | 1.177 | 0.395 | 1.31 | 0.958 | 0.0548 | 1.42 | 0.931 |
| PC aa C34:4 | 0.0488 | 0.236 | 1.183 | - | - | - | 0.0184 | 0.266 | 0.720 |
| PC aa C36:0 | - | - | - | 0.0265 | 0.226 | 0.846 | - | - | - |
| PC aa C36:1 | - | - | - | - | - | - | 0.00345 | 0.682 | 1.20 |
| PC aa C36:2 | 0.0401 | 0.466 | 1.11 | 0.0712 | 2.10 | 0.876 | 0.0548 | 1.70 | 0.925 |
| PC aa C36:3 | 0.419 | 1.78 | 0.988 | - | - | - | - | - | - |
| PC aa C36:4 | - | - | - | - | - | - | 0.458 | 1.02 | 0.981 |
| PC aa C36:5 | - | - | - | 0.0591 | 0.301 | 0.904 | - | - | - |
| PC aa C36:6 | 0.0191 | 0.311 | 1.21 | - | - | - | - | - | - |
| PC aa C38:0 | 0.0649 | 0.144 | 1.17 | - | - | - | - | - | - |
| PC aa C38:4 | - | - | - | 0.00394 | 1.92 | 0.855 | 0.458 | 1.05 | 1.01 |
| PC aa C38:5 | - | - | - | 0.00302 | 0.705 | 0.868 | 0.0887 | 0.586 | 0.950 |
| PC aa C38:6 | - | - | - | 0.0649 | 0.860 | 0.901 | 0.163 | 1.13 | 0.935 |
| PC aa C40:3 | 0.0184 | 0.0651 | 0.867 | 0.00844 | 0.151 | 0.870 | - | - | - |
| PC aa C40:4 | - | - | - | 0.0488 | 0.131 | 0.890 | - | - | - |
| PC aa C40:5 | 0.0712 | 0.238 | 0.934 | 0.0213 | 0.366 | 0.853 | - | - | - |
| PC aa C40:6 | - | - | - | 0.0171 | 0.851 | 0.880 | - | - | - |
| PC aa C42:1 | 0.0327 | 0.0377 | 0.941 | - | - | - | - | - | - |
| PC aa C42:5 | 0.0171 | 0.0508 | 1.15 | - | - | - | 0.0972 | 0.067 | 0.892 |
| PC aa C42:6 | - | - | - | 0.0213 | 0.0595 | 0.831 | - | - | - |
| PC ae C30:1 | 0.0591 | 0.0287 | 0.961 | 0.0401 | 0.109 | 1.20 | - | - | - |
| PC ae C34:1 | - | - | - | 0.00264 | 0.276 | 0.867 | - | - | - |
| PC ae C34:3 | 0.00230 | 0.288 | 1.24 | 0.0930 | 0.0858 | 0.939 | - | - | - |
| PC ae C36:0 | 0.0327 | 0.0767 | 1.12 | 0.00511 | 0.134 | 0.839 | - | - | - |
| PC ae C36:1 | - | - | - | 0.0265 | 0.158 | 0.904 | 0.0291 | 0.109 | 1.09 |
| PC ae C36:2 | - | - | - | - | - | - | 0.0606 | 0.219 | 1.07 |
| PC ae C36:4 | - | - | - | 0.0712 | 0.238 | 0.914 | - | - | - |
| PC ae C36:5 | - | - | - | 0.00302 | 0.232 | 0.886 | - | - | - |
| PC ae C38:1 | 0.0779 | 0.135 | 1.12 | 0.0238 | 0.117 | 0.820 | - | - | - |
| PC ae C38:2 | - | - | - | 0.0591 | 0.185 | 0.898 | 0.0184 | 0.172 | 1.13 |
| PC ae C38:3 | - | - | - | 0.0591 | 0.156 | 0.874 | - | - | - |
| PC ae C38:4 | - | - | - | 0.0852 | 0.228 | 0.936 | - | - | - |
| PC ae C38:5 | - | - | - | 0.00230 | 0.334 | 0.847 | - | - | - |
| PC ae C40:2 | - | - | - | 0.00844 | 0.115 | 0.869 | - | - | - |
| PC ae C40:4 | - | - | - | 0.0191 | 0.177 | 0.934 | - | - | - |
| PC ae C40:5 | - | - | - | 0.0213 | 0.152 | 0.894 | - | - | - |
| PC ae C42:0 | - | - | - | 0.0143 | 0.0782 | 0.822 | - | - | - |
| PC ae C44:3 | 0.000622 | 0.0693 | 0.849 | 0.0451 | 0.0130 | 0.858 | - | - | - |
| PC ae C44:4 | - | - | - | 0.0538 | 0.0461 | 0.901 | - | - | - |
| PC ae C44:6 | - | - | - | 0.0790 | 0.0267 | 0.927 | - | - | - |
| **Sphingolipids (Sphingomyelins [SM]; Hydroxylated Sphingomyelins [SM (OH)])** | | | | | | | | | |
| SM (OH) C14:1 | 0.0362 | 0.0674 | 1.14 | 0.0712 | 0.113 | 0.930 | - | - | - |
| SM (OH) C16:1 | - | - | - | - | - | - | 0.0232 | 0.134 | 0.875 |
| SM (OH) C22:1 | 0.0152 | 0.265 | 1.16 | 0.0107 | 0.334 | 0.866 | - | - | - |
| SM (OH) C22:2 | - | - | - | 0.0488 | 0.235 | 0.863 | - | - | - |
| SM (OH) C24:1 | 0.0930 |  | 1.12 | 0.000975 | 0.166 | 0.820 | - | - | - |
| SM C16:0 | 0.00747 | 0.555 | 1.13 | 0.0443 | 0.636 | 0.893 | 0.00106 | 0.94 | 0.839 |
| SM C16:1 | - | - | - | - | - | - | 0.0402 | 0.415 | 0.856 |
| SM C18:0 | - | - | - | 0.0171 | 0.362 | 0.809 | 0.0402 | 0.341 | 0.891 |
| SM C18:1 | - | - | - | 0.0401 | 0.234 | 0.877 | 0.0184 | 0.346 | 0.824 |
| SM C22:3 | - | - | - | - | - | - | 0.0397 | 0.072 | 0.619 |
| SM C24:0 | - | - | - | 0.0362 | 0.234 | 0.936 | 0.0887 | 0.347 | 0.934 |
| SM C24:1 | - | - | - | 0.0136 | 0.751 | 0.848 | 0.0260 | 0.585 | 0.886 |
| SM C26:0 | 0.0362 | 0.0575 | 1.48 | - | - | - | - | - | - |
| SM C26:1 | 0.0294 | 0.0724 | 1.31 | - | - | - | - | - | - |
| **Metabolite Sums/Rations** |  |  |  |  |  |  |  |  |  |
| (Acetylcarnitine+ Propionylcarnitine) / Carnitine | 0.0152 | NA | 1.07 | 0.0213 | NA | 1.11 | - | - | - |
| (Leucine+ Glutamate) / Glutamine | - | - | - | 0.0852 | NA | 0.857 | 0.0447 | NA | 0.860 |
| Asymmetric dimethylarginine / Arginine | - | - | - | 0.0930 | NA | 1.33 | - | - | - |
| Arginine / (Arginine+Ornithine) | - | - | - | - | - | - | 0.00091 | NA | 1.08 |
| Octadecanoylcarnitine / Octadecenoylcarnitine | - | - | - | 0.0238 | NA | 0.919 | 0.0972 | NA | 0.918 |
| Acetylcarnitine / Carnitine | 0.0191 | NA | 1.0 | 0.0712 | NA | 1.07 | - | - | - |
| Propionylcarnitine / Butyrylcarnitine | 0.0591 | NA | 0.922 | 0.000664 | NA | 1.42 | - | - | - |
| Butyrylcarnitine / Carnitine | 0.0213 | NA | 1.14 | 0.0325 | NA | 1.13 | 0.0149 | NA | 1.23 |
| Butyrylcarnitine / Valerylcarnitine | - | - | - | - | - | - | 0.00835 | NA | 1.44 |
| Citrulline / Arginine | - | - | - | 0.00200 | NA | 1.27 | - | - | - |
| Citrulline / Ornithine | - | - | - | 0.0488 | NA | 1.16 | 0.000086 | NA | 1.46 |
| Carnitine palmitoyltransferase I ratio | - | - | - | 0.0930 | NA | 0.75 | - | - | - |
| Essential Amino Acids | 0.0294 | NA | 1.07 | 0.0852 | NA | 1.10 | 0.0232 | NA | 0.888 |
| Glutamate / Glutamine | - | - | - | 0.0488 | NA | 0.804 | - | - | - |
| Glucogenic Amino Acids | - | - | - | 0.0362 | NA | 1.06 | 0.0447 | NA | 1.13 |
| Glutaminolysis | 0.0930 | NA | 0.898 | - | - | - | - | - | - |
| Glycine / Glutamine | - | - | - | 0.00659 | NA | 0.781 | - | - | - |
| Glycine / Histidine | 0.0294 | NA | 1.17 | - | - | - | - | - | - |
| Glycine / Serine | - | - | - | 0.00449 | NA | 0.844 | 0.0548 | NA | 1.18 |
| Glycolysis | 0.0712 | NA | 0.765 | 0.0362 | NA | 1.06 | 0.0447 | NA | 1.13 |
| lysoPC a C16:0 / lysoPC a C16:1 | - | - | - | - | - | - | 0.0887 | NA | 0.921 |
| lysoPC a C20:4 / lysoPC a C20:3 | - | - | - | - | - | - | 0.0972 | NA | 0.927 |
| Methionine sulfoxide / Methionine | - | - | - | 0.0327 | NA | 1.08 | 0.000123 | NA | 1.27 |
| Monounsaturated Fatty Acids (PC) | 0.0184 | NA | 1.14 | - | - | - | - | - | - |
| Monounsaturated Fatty Acids (PC) / Saturated Fatty Acids (PC) | 0.0890 | NA | 1.06 | - | - | - | 0.0253 | NA | 1.23 |
| Non-essential Amino Acids | 0.00113 | NA | 1.11 | 0.0191 | NA | 1.11 | 0.0100 | NA | 1.12 |
| Ornithine / Arginine | - | - | - | - | - | - | 0.00091 | NA | 0.753 |
| Ornithine / Serine | - | - | - | - | - | - | - | - | - |
| PC aa C36:3 / PC aa C36:4 | - | - | - | - | - | - | 0.0809 | NA | 1.05 |
| PC aa C40:3 / PC aa C42:5 | - | - | - | 0.0238 | NA | 0.894 | - | - | - |
| PC ae C32:1 / PC ae C34:1 | - | - | - | 0.0591 | NA | 1.04 | 0.0260 | NA | 1.05 |
| PC ae C44:5 / PC ae C42:5 | - | - | - | - | - | - | - | - | - |
| Putrescine / Ornithine | - | - | - | 0.0191 | NA | 0.8 | - | - | - |
| Serotonin / Tryptophan | - | - | - | 0.0930 | NA | 0.857 | 0.0972 | NA | 1.18 |
| Tryptophan / Serine | 0.0362 | NA | 1.14 | - | - | - | - | - | - |
| Total Amino Acids | 0.00113 | NA | 1.09 | 0.0121 | NA | 1.130 | 0.0736 | NA | 1.09 |
| Total Sphingomyelins | 0.0649 | NA | 1.07 | 0.0191 | NA | 0.880 | 0.00597 | NA | 0.881 |
| Total Non-Hydroxylated Sphingomyelins | 0.0852 | NA | 1.07 | 0.0238 | NA | 0.891 | 0.00597 | NA | 0.865 |
| Total Hydroxylated Sphingomyelins | 0.0265 | NA | 1.12 | 0.00747 | NA | 0.869 | - | - | - |
| Total Hydroxylated Sphingomyelins / Total Non-Hydroxylated Sphingomyelins | - | - | - | - | - | - | 0.0026 | NA | 1.12 |
