# Supplementary material for: Oral administration of TiO2 nanoparticles during early life impacts cardiac and neurobehavioral performance and metabolite profile in an age- and sex-related manner
Source: Part Fibre Toxicol. 2022 Jan 5;19:3. doi: 10.1186/s12989-021-00444-9 (PMC8728993; doi:10.1186/s12989-021-00444-9)
Supplement: Supplementary file 5 — Additional file 5: Table S3. Metabolites in plasma collected from female pups (n = 14–15) with significant P-value ≤ 0.05 and/or VIP > 1.0 with an S.E. less than mean. Metabolites with a P-value ≤ 0.1 are also shown. [file 12989_2021_444_MOESM5_ESM.docx]

**Supplement Table 3.** Metabolites in plasma collected from female pups (n = 14–15) with significant P-value ≤ 0.05 and/or VIP > 1.0 with an S.E. less than mean. Metabolites with a P-value ≤ 0.1 are also shown.

|  | **PND 2-5** | | | **PND 7-10** | | | **PND 17-20** | | |
| --- | --- | --- | --- | --- | --- | --- | --- | --- | --- |
| **Metabolite** | **p-value** | **VIP** | **fold change** | **p-value** | **VIP** | **fold change** | **p-value** | **VIP** | **fold change** |
| **Amino Acids** |  |  |  |  |  |  |  |  |  |
| Alanine | 0.0294 | 3.35 | 1.09 | 0.0327 | 3.13 | 1.167 | 0.127 | 3.61 | 1.04 |
| Arginine | 0.00659 | 4.21 | 1.46 | - | - | - | 0.206 | 2.46 | 1.08 |
| Asparagine | 0.407 | 1.10 | 0.887 | 0.0238 | 1.15 | 1.22 | - | - | - |
| Citrulline | 0.0213 | 2.80 | 1.25 | - | - | - | 0.0145 | 3.38 | 1.12 |
| Glutamine | 0.00953 | 5.59 | 1.34 | 0.00200 | 6.28 | 1.31 | 0.00345 | 5.85 | 1.16 |
| Glutamate | 0.0538 | 0.475 | 1.10 | - | - | - | - | - | - |
| Glycine | 0.0538 | 3.11 | 1.104 | 0.820 | 1.76 | 0.982 | 0.0736 | 3.31 | 1.18 |
| Histidine | - | - | - | 0.0890 | 0.902 | 1.12 | - | - | - |
| Isoleucine | 0.206 | 0.96 | 0.969 | 0.0238 | 1.40 | 1.18 | 0.458 | 1.47 | 0.917 |
| Leucine | 0.836 | 1.17 | 1.00 | 0.0107 | 2.67 | 1.24 | - | - | - |
| Lysine | 0.351 | 3.29 | 0.917 | 0.0649 | 3.87 | 1.30 | - | - | - |
| Methionine | - | - | - | 0.00151 | 1.76 | 1.19 | 0.0184 | 1.63 | 1.21 |
| Ornithine | 0.00700 | 2.03 | 1.20 | - | - | - | 0.0808 | 1.83 | 0.882 |
| Phenylalanine | - | - | - | 0.0442 | 1.14 | 1.16 | - | - | - |
| Proline | 0.885 | 1.31 | 0.990 | 0.0265 | 2.10 | 1.09 | 0.295 | 1.78 | 1.05 |
| Serine | 0.272 | 1.92 | 1.09 | 0.00511 | 3.47 | 1.33 | - | - | - |
| Threonine | 0.724 | 1.51 | 0.929 | 0.000778 | 2.86 | 1.24 | - | - | - |
| Tryptophan | - | - | - | 0.0464 | 1.09 | 1.06 | - | - | - |
| Tyrosine | - | - | - | 0.198 | 1.0 | 1.073 | 0.162 | 1.05 | 1.05 |
| Valine | 0.772 | 1.11 | 0.991 | 0.0649 | 2.68 | 1.09 | - | - | - |
| **Biogenic Amines** |  |  |  |  |  |  |  |  |  |
| Acetylornithine | - | - | - | 0.000063 | 0.775 | 1.86 | 0.000023 | 0.894 | 1.99 |
| Asymmetric dimethylarginine | 0.0327 | 0.229 | 1.46 | 0.0488 | 0.0463 | 1.30 | - | - | - |
| α-Aminoadipic acid | - | - | - | 0.0488 | 0.114 | 0.770 | - | - | - |
| Histamine | - | - | - | 0.00539 | 0.229 | 1.24 | 0.000213 | 0.458 | 1.77 |
| Kynurenine | - | - | - | 0.0488 | 0.100 | 1.26 | - | - | - |
| Methionine sulfoxide | - | - | - | **0.000025** | 0.680 | **1.65** | 0.000103 | 0.784 | 1.42 |
| Putrescine | **0.000333** | 0.255 | **1.36** | - | - | - | - | - | - |
| Spermidine | - | - | - | - | - | - | 0.0129 | 0.746 | 1.22 |
| Trans-4-Hydroxyproline | - | - | - | - | - | - | 0.00124 | 1.48 | 1.12 |
| **Sugar** |  |  |  |  |  |  |  |  |  |
| Hexoses (including glucose) | 0.885 | 6.19 | 0.984 | 0.310 | 7.36 | 1.03 | 0.222 | 7.13 | 0.978 |
| **Acylcarnitines** |  |  |  |  |  |  |  |  |  |
| Carnitine | - | - | - | 0.00953 | 0.690 | 1.22 | - | - | - |
| Acetylcarnitine | - | - | - | 0.00747 | 0.373 | 1.13 | 0.0026 | 0.516 | 1.27 |
| Propionylcarnitine | - | - | - | 0.000246 | 0.140 | 1.50 | 0.0668 | 0.0559 | 1.05 |
| Butyrylcarnitine | - | - | - | 0.0226 | 0.106 | 1.22 | 0.00115 | 0.138 | 1.39 |
| Valerylcarnitine | - | - | - | 0.00458 | 0.0325 | 1.25 | 0.0176 | 0.0189 | 1.16 |
| Hydroxyvalerylcarnitine (Methylmalonylcarnitine) | - | - | - | 0.0317 | 0.0260 | 1.33 | - | - | - |
| Hexanoylcarnitine (Fumarylcarnitine) | - | - | - | 0.0641 | 0.00967 | 0.770 | - | - | - |
| Nonaylcarnitine | - | - | - | - | - | - | 0.0393 | 0.0438 | 1.17 |
| Tetradecanoylcarnitine | - | - | - | 0.0890 | 0.0138 | 0.761 | - | - | - |
| Tetradecenoylcarnitine | 0.0330 | 0.0388 | 0.896 | 0.00372 | 0.0416 | 0.787 | - | - | - |
| Hexadecanoylcarnitine | - | - | - | 0.00449 | 0.0484 | 0.789 | - | - | - |
| Octadecanoylcarnitine | 0.0121 | 0.0409 | 0.848 | 0.00507 | 0.0313 | 0.860 | - | - | - |
| Octadecenoylcarnitine | - | - | - | 0.00449 | 0.0331 | 0.811 | - | - | - |
| **Glycerophospholipids (Lysophosphatidylcholines [lysoPC]; Phosphatidylcholines [PC])** | | | | | | | | | |
| lysoPC a C16:0 | 0.120 | 1.02 | 0.967 | - | - | - | - | - | - |
| lysoPC a C16:1 | - | - | - | 0.0538 | 0.140 | 1.12 | 0.0207 | 0.243 | 1.16 |
| lysoPC a C17:0 | - | - | - | 0.0121 | 0.112 | 1.16 | 0.0207 | 0.155 | 1.16 |
| lysoPC a C18:0 | 0.0213 | 1.22 | 0.907 | - | - | - | 0.0548 | 0.716 | 1.11 |
| lysoPC a C18:2 | 0.120 | 1.12 | 0.896 | 0.0443 | 0.679 | 1.16 | - | - | - |
| lysoPC a C20:4 | 0.00747 | 0.612 | 0.903 | - | - | - | - | - | - |
| lysoPC a C24:0 | - | - | - | - | - | - | 0.0424 | 0.225 | 1.04 |
| lysoPC a C26:0 | - | - | - | 0.0433 | 0.0430 | 0.928 | - | - | - |
| lysoPC a C28:0 | - | - | - | - | - | - | 0.0115 | 0.123 | 1.45 |
| PC aa C26:0 | - | - | - | - | - | - | 0.0914 | 0.172 | 1.17 |
| PC aa C30:0 | - | - | - | - | - | - | 0.0325 | 0.237 | 0.853 |
| PC aa C32:0 | - | - | - | - | - | - | 0.0145 | 0.446 | 0.887 |
| PC aa C32:2 | - | - | - | 0.0538 | 0.254 | 1.36 | - | - | - |
| PC aa C32:3 | - | - | - | 0.0488 | 0.0379 | 1.20 | - | - | - |
| PC aa C34:2 | 0.0121 | 2.25 | 0.881 | - | - | - | - | - | - |
| PC aa C34:3 | - | - | - | 0.0265 | 0.275 | 1.22 | - | - | - |
| PC aa C34:4 | 0.0852 | 0.24 | 0.774 | - | - | - | 0.0736 | 0.124 | 0.923 |
| PC aa C36:0 | 0.000333 | 0.295 | 0.843 | - | - | - | - | - | - |
| PC aa C36:1 | - | - | - | - | - | - | 0.0291 | 0.633 | 1.21 |
| PC aa C36:2 | 0.00264 | 2.72 | 0.831 | - | - | - | 0.0548 | 2.50 | 0.863 |
| PC aa C36:3 | 0.00747 | 1.17 | 0.905 | - | - | - | - | - | - |
| PC aa C36:4 | 0.000125 | 2.13 | 0.883 | - | - | - | 0.0887 | 1.02 | 0.924 |
| PC aa C38:0 | 0.00581 | 0.229 | 0.895 | - | - | - | - | - | - |
| PC aa C38:1 | 0.0401 | 0.128 | 0.782 | - | - | - | - | - | - |
| PC aa C38:3 | 0.0443 | 0.569 | 0.889 | - | - | - | - | - | - |
| PC aa C38:4 | 0.000037 | 2.75 | 0.770 | - | - | - | - | - | - |
| PC aa C38:5 | 0.00264 | 0.880 | 0.819 | - | - | - | - | - | - |
| PC aa C38:6 | 0.00264 | 1.42 | 0.890 | - | - | - | 0.0402 | 0.910 | 0.847 |
| PC aa C40:1 | 0.0248 | 0.128 | 0.881 | - | - | - | 0.0545 | 0.0429 | 0.865 |
| PC aa C40:3 | 0.0779 | 0.13 | 0.898 | - | - | - | 0.0809 | 0.147 | 0.830 |
| PC aa C40:4 | 0.000015 | 0.281 | 0.788 | - | - | - | - | - | - |
| PC aa C40:5 | 0.0265 | 0.352 | 0.803 | - | - | - | - | - | - |
| PC aa C40:6 | 0.000533 | 1.27 | 0.856 | - | - | - | - | - | - |
| PC aa C42:0 | 0.0145 | 0.0333 | 0.895 | - | - | - | - | - | - |
| PC aa C42:1 | - | - | - | - | - | - | 0.0260 | 0.0762 | 1.153 |
| PC aa C42:5 | 0.000391 | 0.110 | 0.881 | 0.0488 | 0.0690 | 0.905 | 0.0291 | 0.0585 | 0.852 |
| PC aa C42:6 | 0.000125 | 0.121 | 0.874 | - | - | - | - | - | - |
| PC ae C30:1 | - | - | - | 0.0538 | 0.0392 | 1.57 | 0.0447 | 0.0636 | 1.22 |
| PC ae C34:1 | - | - | - | 0.0712 | 0.147 | 0.917 | - | - | - |
| PC ae C34:2 | - | - | - | 0.000975 | 0.252 | 1.19 | - | - | - |
| PC ae C36:0 | 0.00953 | 0.141 | 0.887 | 0.0171 | 0.106 | 0.852 | - | - | - |
| PC ae C36:1 | 0.0591 | 0.140 | 0.877 | - | - | - | - | - | - |
| PC ae C36:2 | 0.0712 | 0.328 | 0.960 | 0.00174 | 0.321 | 1.16 | - | - | - |
| PC ae C36:3 | - | - | - | 0.0294 | 0.128 | 1.08 | 0.0972 | 0.148 | 1.05 |
| PC ae C36:4 | 0.0538 | 0.354 | 0.966 | - | - | - | - | - | - |
| PC ae C36:5 | 0.00581 | 0.253 | 0.876 | - | - | - | - | - | - |
| PC ae C38:0 | - | - | - | 0.0852 | 0.10671 | 1.13 | - | - | - |
| PC ae C38:1 | 0.00345 | 0.221 | 0.773 | - | - | - | - | - | - |
| PC ae C38:2 | 0.00151 | 0.317 | 0.858 | - | - | - | - | - | - |
| PC ae C38:3 | 0.00200 | 0.214 | 0.814 | - | - | - | - | - | - |
| PC ae C38:4 | 0.0265 | 0.325 | 0.888 | - | - | - | - | - | - |
| PC ae C38:5 | 0.00131 | 0.450 | 0.880 | - | - | - | - | - | - |
| PC ae C38:6 | 0.00844 | 0.305 | 0.894 | - | - | - | 0.0972 | 0.212 | 0.876 |
| PC ae C40:1 | 0.00659 | 0.242 | 0.830 | 0.0152 | 0.174 | 1.23 | - | - | - |
| PC ae C40:2 | 0.00581 | 0.122 | 0.910 | - | - | - | - | - | - |
| PC ae C40:3 | 0.00223 | 0.131 | 0.900 | - | - | - | - | - | - |
| PC ae C40:4 | 0.000333 | 0.263 | 0.835 | - | - | - | - | - | - |
| PC ae C40:5 | 0.00230 | 0.206 | 0.887 | - | - | - | - | - | - |
| PC ae C40:6 | 0.0107 | 0.233 | 0.910 | - | - | - | - | - | - |
| PC ae C42:0 | 0.006132 | 0.221 | 0.848 | - | - | - | 0.00686 | 0.0729 | 0.901 |
| PC ae C42:1 | 0.000008 | 0.184 | 0.755 | - | - | - | 0.00124 | 0.119 | 1.17 |
| PC ae C42:2 | 0.000391 | 0.138 | 0.851 | - | - | - | - | - | - |
| PC ae C42:3 | 0.00511 | 0.174 | 0.845 | 0.0488 | 0.0952 | 1.22 | - | - | - |
| PC ae C42:5 | - | - | - | - | - | - | 0.0641 | 0.0646 | 1.22 |
| PC ae C44:3 | 0.00151 | 0.0698 | 0.826 | - | - | - | - | - | - |
| PC ae C44:4 | 0.000724 | 0.0885 | 0.797 | 0.0401 | 0.0502 | 0.832 | - | - | - |
| PC ae C44:6 | 0.00394 | 0.0526 | 0.862 | - | - | - | - | - | - |
| **Sphingolipids (Sphingomyelins [SM]; Hydroxylated Sphingomyelins [SM (OH)])** | | | | | | | | | |
| SM (OH) C16:1 | - | - | - | - | - | - | 0.0232 | 0.122 | 0.896 |
| SM (OH) C24:1 | - | - | - | 0.0712 | 0.060 | 0.904 | - | - | - |
| SM C18:0 | - | - | - | - | - | - | 0.00194 | 0.637 | 0.891 |
| SM C18:1 | 0.00345 | 0.364 | 0.852 | - | - | - | 0.00597 | 0.380 | 0.844 |
| SM C22:3 | - | - | - | 0.0990 | 0.0471 | 2.02 | - | - | - |
| SM C24:0 | 0.0488 | 0.428 | 0.923 | - | - | - | - | - | - |
| SM C24:1 | 0.00953 | 0.638 | 0.897 | - | - | - | - | - | - |
| SM C26:0 | 0.0443 | 0.0575 | 1.22 | - | - | - | - | - | - |
| **Metabolite Sums/Rations** |  |  |  |  |  |  |  |  |  |
| (Acetylcarnitine+ Propionylcarnitine) / Carnitine | - | - | - | - | - | - | 0.00522 | NA | 1.17 |
| (Leucine+ Glutamate) / Glutamine | 0.0213 | NA | 0.760 | 0.0930 | NA | 0.917 | 0.0129 | NA | 0.802 |
| Asymmetric dimethylarginine / Arginine | - | - | - | 0.0930 | NA | 1.33 | - | - | - |
| Arginine / (Arginine+Ornithine) | - | - | - | - | - | - | 0.0232 | NA | 1.09 |
| Branched-Chain Amino Acids | - | - | - | 0.0213 | NA | 1.19 | - | - | - |
| Octadecanoylcarnitine / Octadecenoylcarnitine | 0.0362 | NA | 0.895 | - | - | - | - | - | - |
| Acetylcarnitine / Carnitine | - | - | - | - | - | - | 0.00456 | NA | 1.16 |
| Propionylcarnitine / Butyrylcarnitine | - | - | - | 0.0322 | NA | 1.20 | 0.00190 | NA | 0.832 |
| Butyrylcarnitine / Carnitine | 0.0538 | NA | 1.13 | - | - | - | 0.00308 | NA | 1.23 |
| Butyrylcarnitine / Valerylcarnitine | - | - | - | 0.0750 | NA | 0.870 | - | - | - |
| Citrulline / Ornithine | - | - | - | - | - | - | 0.0026 | NA | 1.31 |
| Carnitine palmitoyltransferase I ratio | - | - | - | 0.00111 | NA | 0.7 | - | - | - |
| Essential Amino Acids | - | - | - | 0.00953 | NA | 1.20 | - | - | - |
| Glutamate / Glutamine | 0.0779 | NA | 0.876 | 0.00345 | NA | 0.772 | 0.0402 | NA | 0.779 |
| Glucogenic Amino Acids | 0.0213 | NA | 1.12 | 0.0649 | NA | 1.10 | 0.0736 | NA | 1.08 |
| Glutaminolysis | 0.0265 | NA | 0.907 | - | - | - | 0.0668 | NA | 0.897 |
| Glycine / Arginine | 0.0779 | NA | 0.902 | - | - | - | - | - | - |
| Glycine / Glutamine | - | - | - | 0.00151 | NA | 0.821 | - | - | - |
| Glycine / Histidine | - | - | - | - | - | - | 0.0184 | NA | 1.18 |
| Glycine / Serine | - | - | - | 0.0152 | NA | 0.833 | 0.0362 | NA | 1.09 |
| Glycolysis | 0.0213 | NA | 1.12 | 0.0649 | NA | 1.10 | 0.07355 | NA | 1.08 |
| lysoPC a C16:0 / lysoPC a C16:1 | - | - | - | - | - | - | 0.0129 | NA | 0.885 |
| lysoPC a C20:4 / lysoPC a C20:3 | - | - | - | 0.00844 | NA | 0.849 | 0.0291 | NA | 0.900 |
| Methionine sulfoxide / Methionine | - | - | - | 0.000333 | NA | 1.25 | 0.000292 | NA | 1.28 |
| Non-essential Amino Acids | 0.00174 | NA | 1.20 | 0.00844 | NA | 1.13 | 0.0548 | NA | 1.11 |
| Ornithine / Arginine | - | - | - | - | - | - | 0.0232 | NA | 0.747 |
| Ornithine / Serine | - | - | - | 0.0779 | NA | 0.849 | 0.0736 | NA | 0.787 |
| PC aa C36:3 / PC aa C36:4 | - | - | - | 0.0171 | NA | 1.08 | 0.0668 | NA | 1.05 |
| PC aa C40:3 / PC aa C42:5 | - | - | - | - | - | - | - | - | - |
| PC ae C32:1 / PC ae C34:1 | - | - | - | - | - | - | 0.0260 | NA | 1.07 |
| PC ae C44:5 / PC ae C42:5 | - | - | - | - | - | - | 0.0641 | NA | 0.712 |
| Putrescine / Ornithine | - | - | - | 0.0265 | NA | 0.875 | - | - | - |
| Saturated Fatty Acids (PC) | 0.0896 | NA | 0.927 | - | - | - | - | - | - |
| Spermidine / Putrescine | 0.0265 | NA | 0.804 | - | - | - | 0.0145 | NA | 1.28 |
| Spermine / Spermidine | 0.0930 | NA | 0.936 | - | - | - | 0.00456 | NA | 0.863 |
| Tryptophan / Serine | - | - | - | - | - | - | - | - | - |
| Total Amino Acids | 0.00581 | NA | 1.10 | 0.00131 | NA | 1.14 | 0.0888 | NA | 1.07 |
| Total PC aa | 0.0201 | NA | 0.905 | - | - | - | - | - | - |
| Total Sphingomyelins | 0.0649 | NA | 0.952 | - | - | - | - | - | - |
| Total Non-Hydroxylated Sphingomyelins | 0.0591 | NA | 0.947 | - | - | - | - | - | - |
| Total Hydroxylated Sphingomyelins / Total Non-Hydroxylated Sphingomyelins | - | - | - | - | - | - | 0.0362 | NA | 1.12 |
| Tyrosine / Phenylalanine | 0.0852 | NA | 1.10 | - | - | - | - | - | - |
