# Supplementary material for: Oral administration of TiO2 nanoparticles during early life impacts cardiac and neurobehavioral performance and metabolite profile in an age- and sex-related manner
Source: Part Fibre Toxicol. 2022 Jan 5;19:3. doi: 10.1186/s12989-021-00444-9 (PMC8728993; doi:10.1186/s12989-021-00444-9)
Supplement: Supplementary file 6 — Additional file 6: Table S4. Chemical formulation of digestion of in vitro rat gastrointestinal solutions tested in this study and time (h) of digestions. [file 12989_2021_444_MOESM6_ESM.docx]

**Supplement Table 4.** Chemical formulation of digestion of *in vitro* rat gastrointestinal solutions tested in this study and time (h) of digestions.

| **Solution** | **Bland phase**  **(~PND 7)** | **Transitional phase (~PND 14)** | **Acidic phase**  **(~PND 21)** |
| --- | --- | --- | --- |
| **Chemical formulation** | 0.315 g/L NaHCO_3_  8.78 g/L NaCl | 0.13 g/L Pepsin (from porcine gastric mucosa)  1.5 g/L Mucin  Hydrochloric acid  0.315 g/L NaHCO_3_  8.78 g/L NaCl | 0.27 g/L Pepsin (from porcine gastric mucosa)  1.5 g/L Mucin  Hydrochloric acid  0.315 g/L NaHCO_3_  8.78 g/L NaCl |
| **pH** | 7.0 | 6.0 | 4.0 |
| **Time points** | 1, 2, and 4 hours | 1, 2, and 4 hours | 1, 2, and 4 hours |
